# Indolepropionic Acid, a Metabolite of the Microbiome, Has Cytostatic Properties in Breast Cancer by Activating AHR and PXR Receptors and Inducing Oxidative Stress

**DOI:** 10.3390/cancers12092411

**Published:** 2020-08-25

**Authors:** Zsanett Sári, Edit Mikó, Tünde Kovács, Laura Jankó, Tamás Csonka, Gréta Lente, Éva Sebő, Judit Tóth, Dezső Tóth, Péter Árkosy, Anita Boratkó, Gyula Ujlaki, Miklós Török, Ilona Kovács, Judit Szabó, Borbála Kiss, Gábor Méhes, James J. Goedert, Péter Bai

**Affiliations:** 1 Department of Medical Chemistry, Faculty of Medicine, University of Debrecen, 4032 Debrecen, Hungary; sari.zsanett@med.unideb.hu (Z.S.); miko.edit@med.unideb.hu (E.M.); kovacs.tunde@med.unideb.hu (T.K.); janko.laura90@gmail.com (L.J.); lentegreti@gmail.com (G.L.); boratko@med.unideb.hu (A.B.); ujlakigyula15@gmail.com (G.U.); 2 MTA-DE Lendület Laboratory of Cellular Metabolism, 4032 Debrecen, Hungary; 3 Department of Pathology, Faculty of Medicine, University of Debrecen, 4032 Debrecen, Hungary; csonkatamas84@gmail.com (T.C.); gabor.mehes@med.unideb.hu (G.M.); 4 Kenézy Breast Center at Kenézy Gyula County Hospital, 4032 Debrecen, Hungary; seboeva@gmail.com; 5 Department of Oncology, Faculty of Medicine, University of Debrecen, 4032 Debrecen, Hungary; tothjuditdr11@t-online.hu (J.T.); arkosy.peter@med.unideb.hu (P.Á.); bkiss@med.unideb.hu (B.K.); 6 Department of Surgery, Borsod-Abaúj-Zemplén County Hospital and University Teaching Hospital, 3526 Miskolc, Hungary; detoth@gmail.com; 7 Department of Pathology at Kenézy Gyula County Hospital, 4032 Debrecen, Hungary; dr.torok.miklos@kenezy.unideb.hu (M.T.); dr.kovacs.ilona@kenezy.unideb.hu (I.K.); 8 Department of Medical Microbiology, Faculty of Medicine, University of Debrecen, 4032 Debrecen, Hungary; szabjud@med.unideb.hu; 9 National Cancer Institute, National Institutes of Health, Bethesda, MD 20982, USA; goedertj@mail.nih.gov; 10 Research Center for Molecular Medicine, Faculty of Medicine, University of Debrecen, 4032 Debrecen, Hungary

**Keywords:** breast cancer, microbiome, oncobiome, indolepropionic acid, AHR, PXR, metastasis, oxidative stress, nitrosative stress, epithelial-to-mesenchymal transition

## Abstract

Oncobiotic transformation of the gut microbiome may contribute to the risk of breast cancer. Recent studies have provided evidence that the microbiome secretes cytostatic metabolites that inhibit the proliferation, movement, and metastasis formation of cancer cells. In this study, we show that indolepropionic acid (IPA), a bacterial tryptophan metabolite, has cytostatic properties. IPA selectively targeted breast cancer cells, but it had no effects on non-transformed, primary fibroblasts. In cell-based and animal experiments, we showed that IPA supplementation reduced the proportions of cancer stem cells and the proliferation, movement, and metastasis formation of cancer cells. These were achieved through inhibiting epithelial-to-mesenchymal transition, inducing oxidative and nitrosative stress, and boosting antitumor immune response. Increased oxidative/nitrosative stress was due to the IPA-mediated downregulation of nuclear factor erythroid 2-related factor 2 (NRF2), upregulation of inducible nitric oxide synthase (iNOS), and enhanced mitochondrial reactive species production. Increased oxidative/nitrosative stress led to cytostasis and reductions in cancer cell stem-ness. IPA exerted its effects through aryl hydrocarbon receptor (AHR) and pregnane X receptor (PXR) receptors. A higher expression of PXR and AHR supported better survival in human breast cancer patients, highlighting the importance of IPA-elicited pathways in cytostasis in breast cancer. Furthermore, AHR activation and PXR expression related inversely to cancer cell proliferation level and to the stage and grade of the tumor. The fecal microbiome’s capacity for IPA biosynthesis was suppressed in women newly diagnosed with breast cancer, especially with stage 0. Bacterial indole biosynthesis showed correlation with lymphocyte infiltration to tumors in humans. Taken together, we found that IPA is a cytostatic bacterial metabolite, the production of which is suppressed in human breast cancer. Bacterial metabolites, among them, IPA, have a pivotal role in regulating the progression but not the initiation of the disease.

## 1. Introduction

Dysbiotic microbiota, associated with oncological diseases, is termed the oncobiome. Oncobiosis of the distal gut is associated with breast cancer [1,2,3,4,5,6,7,8,9,10,11]; importantly, among the oncobiome studies, common microbiome changes were identified [12,13], suggesting a common root for the pathogenic role of the oncobiome. It remained a question of whether oncobiotic transformation of the microbiome has a causative role in carcinogenesis in breast cancer or if it is an epiphenomenon. Perturbing the microbiota by antibiotic treatment exacerbated breast cancer in an animal model [14], and there is suggestive supportive evidence in human population studies [15,16,17,18,19,20,21,22]. In good agreement with that, probiotic treatment may be protective against the incidence of breast cancer [23,24,25,26]. Taken together, the oncobiome could have a causative role in carcinogenesis.

While there have been several studies of potential oncobiosis in breast cancer, much less is known about how oncobiosis affects breast cancer cells and the pathways by which it may contribute to carcinogenesis. The oncobiome changes the behavior of breast cancer cells at multiple points. Change from eubiosis to oncobiosis induces epithelial-to-mesenchymal transition (EMT) [27,28,29]; promotes migration and invasion [28]; reduces oxidative stress [30]; induces widespread metabolic alterations [27,28] in cancers cells; alters the differentiation of cancer stem cells [28]; and suppresses antitumor immunity [27,31]. These oncogenic processes support tumor growth [27,28], tumor infiltration to the surrounding tissues [27,28], and metastasis formation [27,28,31,32]. Moreover, the oncobiome has enhanced capacity to increase systemic estrogen levels by increasing enterohepatic circulation [1,2,5]. The parent estrogens, estrone and estradiol, increase cell proliferation, and their catechol–quinone metabolites cause oxidative DNA damage and mutagenesis [33]. The microbiome also can interfere with the chemoradiotherapy and immunotherapy of breast cancer [34,35,36].

The actual molecular mechanisms through which the microbiome and cancer cells interact are yet not characterized in detail. Nevertheless, there are two major routes identified to date. The physical presence of the bacteria and their physical interaction with immune cells fine-tune the immune system that directly impacts on the efficacy of immune therapies and antitumor immunity [20,35,37,38]. Additionally, bacterial metabolites can provide a link between the microbiome and cancer cells through the circulation [34]. Among these metabolites, short-chain fatty acids, lithocholic acid, and cadaverine have cytostatic properties [27,28,34,39]. In breast cancer, the metabolic capacity of the microbiome is suppressed [4,27], suggesting that the production of cytostatic metabolites decrease in breast cancer.

Indolepropionic acid (IPA) is a bacterial metabolite that is produced by the deamination of tryptophan [28,40]. The enzyme responsible for the formation of indolepropionic acid is tryptophanase (TnaA) [40]. Approximately, 4–6% of tryptophan is metabolized to indole derivatives through bacterial enzymes [41]. In germ-free mice, serum tryptophan levels increase, highlighting the burden of microbial degradation on tryptophan levels [42,43,44,45,46]. It is of note that besides the indicated indole derivatives, the microbiome is capable of synthesizing a large set of other indole-derivative metabolites [46,47]. In the pathway that yields IPA, tryptophan is converted to indole lactate, then to indole acrylic acid, and subsequently to IPA [48]. In this study, we assessed the cytostatic properties of a bacterial metabolite, IPA, as well as changes to its production in women with breast cancer.

## 2. Materials and Methods

All methods were performed according to the relevant guidelines.

### 2.1. Chemicals

Chemicals, among them, indolepropionic acid (IPA), glutathione (GSH), N-acetyl-cysteine (NAC), Mito-TEMPO, and ketoconazole were from Sigma-Aldrich (St. Louis, MO, USA) unless otherwise stated. IPA was used, 0.4 μM and 0.8 μM, which corresponds to the normal human serum concentration of IPA [40,49,50]. GSH and NAC antioxidants were used at a final concentration of 5 mM. The mitochondria-targeted antioxidant Mito-TEMPO was used at a concentration of 5 μM. The aryl hydrocarbon receptor (AHR) inhibitor, CH223191, was obtained from MedChemExpress (MCE, Monmouth Junction, NJ, USA) and was applied at a concentration of 10 μM. Pregnane X receptor (PXR) downstream signaling was inhibited using ketoconazole at a final concentration of 25 μM [51,52]. The Silencer Select siRNAs targeting AHR (AHR—siRNA ID: s1198) and PXR (NR1I2—siRNA ID: s16910) and the negative control siRNA #1 (cat.no. 4390843) were obtained from Thermo Fisher Scientific (Waltham, MA, USA) and each was used at a final concentration of 30 nM.

### 2.2. Cell Culture

**4T1** murine breast cancer cells were maintained in RPMI-1640 (Sigma-Aldrich, R5886) medium containing 10% FBS, 1% penicillin/streptomycin, 2 mM L-glutamine, and 1% pyruvate at 37 °C with 5% CO_2_.

**SKBR-3** human breast cancer cells were maintained in DMEM (Sigma-Aldrich, 1000 mg/L glucose, D5546) medium containing 10% FBS, 1% penicillin/streptomycin, 2 mM L-glutamine at 37 °C with 5% CO_2_.

**Human primary fibroblasts** cells were maintained in DMEM (Sigma-Aldrich, 1000 mg/L glucose, D5546) medium containing 20% FBS, 1% penicillin/streptomycin, 2 mM L-glutamine at 37 °C with 5% CO_2_.

### 2.3. In Vitro Cell Proliferation Assays

Cellular proliferation was assessed using sulforhodamine B (SRB) assay and colony-forming assay as described in [53]. Cells were seeded in 96-well plates (4T1—1500 cells/well; MDA-MB-231—3000 cells/well; SKBR-3—5000 cells/well; MCF7—4000 cells/well; human fibroblast—7500 cells/well) and were treated with the indicated concentrations of IPA or Mito-TEMPO mitochondria-targeted antioxidant (5 μM) at the presence of IPA (0.8 μM) for 24 h. Then, cells were fixed in 50% trichloroacetic acid (TCA—final concentration: 10%), and the plates were incubated for 1 h at 4 °C. Plates were washed 5 times with water and stained with 0.4% (*w*/*v*) sulforhodamine B solution in 1% acetic acid. Unbound dye was removed by washing 5 times with 1% acetic acid. A bound stain was solubilized with 10 mM Tris base, and the absorbance was measured on an automated plate reader (Thermo Labsystems Multiskan MS, Walthman, MA, USA) at 540 nm.

For colony-forming assays, cells were seeded in a 6-well plate (4T1—500 cells/well; MCF7—1000 cells/well; SKBR-3—1500 cells/well) in complete medium and were cultured with the indicated concentrations of IPA for 7 days. After treatment, the plates were washed twice with PBS. Colonies were fixed in methanol for 15 min, dried, and stained with May–Grünwald–Giemsa solution for 20 min. Cells were washed with water, and the colonies were determined using Image J software (Image J2 version) [54].

### 2.4. Detection of Cell Death

IPA-induced cytotoxicity was assessed by propidium iodide (PI; Biotium, Fremont, CA, 40016, USA) uptake assays as in [55]. Cells were seeded in a 6-well plate (4T1—75,000 cells/well; MCF7—150,000 cells/well; SKBR-3—200,000 cells/well; human fibroblast—200,000 cells/well) and treated with the indicated concentrations of IPA for 24 h; then, they were stained with 100 μg/mL PI for 30 min at 37 °C, washed once in PBS, and analyzed by flow cytometry (FACS Calibur, BD Biosciences).

To assess changes in apoptotic and necrotic cell death, we used an Annexin V+PI double staining assay kit (Invitrogen, OR, USA, V13242). Cells were seeded in 6-well plates (4T1—75,000 cells/well; MCF7—150,000 cells/well; SKBR-3—200,000 cells/well; human fibroblast—200,000 cells/well) treated with the indicated IPA concentrations for 24 h. Then, cells were stained with 100 μg/mL PI solution and 5 μL FITC Annexin V according to the manufacturer’s instructions. The number of apoptotic and necrotic cells were counted using a FacsCalibur flow cytometer (Beckton-Dickinson Franklin Lakes, NJ, USA).

### 2.5. Electric Cell–Substrate Impedance Sensing (ECIS)

ECIS (Electric cell–substrate impedance sensing) measurements (ECIS model Zθ, Applied BioPhysics Inc., Troy, NY, USA) were used to monitor cell-to-cell and cell-to-surface connections. 4T1 cells were seeded (40,000 cells/well) on type 8W10E arrays. After 20 h, cells were treated with vehicle, 0.4 μM, or 0.8 μM IPA, and total impedance values were measured for 24 h. Multifrequency measurements were taken at 62.5, 125, 250, 500, 1000, 2000, 4000, 8000, 16,000, 32,000 and 64,000 Hz. The reference well was set to a no-cell control with complete medium. ECIS assays were performed similarly to [27].

### 2.6. Immunocytochemisry

Immunocytochemistry was performed similarly to [27]. 4T1 cells were grown on glass coverslips for 1 day and treated with the indicated concentrations of IPA for 24 h. Then, the cells were washed in PBS and fixed with 4% paraformaldehyde for 15 min and permeabilized using 1% Triton X-100 in PBS for 5 min. Then, cells were blocked with 1% bovine serum albumin (BSA) in PBS for 1 h and incubated with TexasRed-X Phalloidin (T7471, 1:150, Thermore Fisher, Waltham, MA, USA) for 1 h at 4 °C. Cells nuclei were visualized with DAPI (R37606, 1:10, Thermo Fischer Scientific Inc., Rockford, IL, USA) and rinsed in phosphate buffered saline (PBS) twice for 10 min. Coverslips mounted in Mowiol/Dabco solution. Confocal images were acquired with a Leica TCS SP8 confocal microscope and were processed using LAS AFv3.1.3 software. Typical mesenchymal-like and epithelial-like morphology of 4T1 cells are shown in [27].

### 2.7. mRNA Preparation and Quantitation

Reverse transcription-coupled PCR (RT-qPCR) was performed similarly to [56]. Total RNA from cells were isolated using TRIzol reagent according to the manufacturer’s instructions (Invitrogen Corporation, Carlsbad, CA, USA). For the assessment of the expression of indicated genes, 2 μg of RNA was reverse transcribed using a High-Capacity cDNA Reverse Transcription Kit (Applied Biosystems, Foster City, CA, USA). The qPCR reactions were performed with the qPCRBIO syGreen Lo-ROX Supermix (PCR Biosystems Ltd., London, UK) on a Light-Cycler 480 Detection System (Roche Applied Science, Basel, Switzerland). Gene expression was normalized to the geometric mean of human 36B4 and cyclophyllin values. Primers are listed in Table 1.

### 2.8. Bacterial TnaA DNA Quantitation

The human fecal DNA library from breast cancer patients and matched control women was published in [3]. For the assessment of the abundance of bacterial tryptophanase (TnaA) coding DNA in human fecal, DNA samples consisting of 10 ng of DNA were used for qPCR reactions. Primers are listed in Table 2.

### 2.9. Quantitation of Fecal *E. coli* TnaA Protein

Fecal proteins were isolated as described in [28] and [57]. Fecal samples (100 mg) were lysed in 500 µL RIPA buffer (50 mM Tris, 150 mM NaCl, 0.1% SDS, 1% Triton X-100, 0.5% sodium deoxycholate, 1 mM EDTA, 1 mM Na_3_VO_4_, 1 mM phenylmethylsulfonyl fluoride (PMSF), 1 mM NaF, protease inhibitor cocktail), and sonicated (Qsonica Q125 Sonicator, Newtown, Connecticut) 3 times for 30 s with 50% amplitude. After centrifugation, 8 µL β-mercaptoethanol and 25 µL 5 X SDS sample buffer (50% glycerol, 10% SDS, 310 mM Tris HCl, pH 6.8, 100 mM dithiotreitol (DTT), 0.01% bromophenol blue) were added to each 100 µL extract. Then, fecal protein samples were heated for 10 min at 96 °C and held on ice until loading. Protein extract (40 µL) was loaded on 8% SDS-PAGE gels, and proteins were separated and transferred onto nitrocellulose membrane. Ponceau-red staining took place after transfer, but before blocking. Ponceau-stained membranes were photographed and used in subsequent analyses. Membranes were cut at the 70 kDa standard, and the top part was blotted for TnaA (*E. coli* TnaA, 1:2000, Assaypro (33517–05111)). Membranes were blocked in Tris buffered saline-Tween 20 (TBST) containing 5% BSA for 1 h and incubated with anti-LdcC primary antibody overnight at 4 °C. After washing with 1 x TBS-TWEEN solution, the membranes were probed with peroxidase-conjugated IgG antibodies (1:2000, Cell Signaling Technology, Inc., Beverly, MA, USA). Bands were visualized by enhanced chemiluminescence reaction (SuperSignal West Pico Solutions, Thermo Fisher Scientific Inc., Rockford, IL, USA). Blots were evaluated by densitometry using Image J software, and antibody signals were normalized to total protein stained by Ponceau-red (Sigma-Aldrich).

### 2.10. Aldefluor Assay

Aldehyde dehydrogenase (ALDH) activity was measured using an Aldefluor Stem Cell kit (StemCell Technologies, Vancouver, BC, Canada). 4T1 cells were seeded on 6-well plates (4T1—100,000 cells/well) and treated with different concentrations of IPA for 24 h. Then, cells were collected and prepared according to the manufacturer’s instructions. For positive control samples, the SKBR-3 cell line was used based on the manufacturers’ instructions. Changes in the level of ALDH were determined by flow cytometry, and the results were analyzed by using Flowing Software 2.5.1. Aldefluor assay for assessing stemness was performed similarly to [28,58,59].

### 2.11. SDS-PAGE and Western Blotting

SDS PAGE and Western blotting was performed as in [60]. Cells were lysed in RIPA buffer (50 mM Tris, 150 mM NaCl, 0.1% SDS, 1% TritonX 100, 0.5% sodium deoxycolate, 1 mM EDTA, 1 mM Na_3_VO_4_, 1 mM PSMF, 1 mM NaF, protease inhibitor cocktail). Protein extracts (20–50 μg) were separated on 10% SDS polyacrylamide gels and blotted onto nitrocellulose membranes. Then, membranes were blocked in TBST containing 5% BSA for 1 h and incubated with primary antibodies overnight at 4 °C. After washing with 1 × TBST solution, the membranes were probed with IgG HRP-conjugated peroxidase secondary antibodies (1:2000, Cell Signaling Technology, Inc., Beverly, MA, USA). Bands were visualized by enhanced chemiluminescence reaction (SuperSignal West Pico Solutions, Thermo Fisher Scientific Inc., Rockford, IL, USA). Blots were quantified by densitometry using the Image J software. Densitometric analysis is represented as a separate supplementary file, and it is also uploaded among the primary data. The primary and secondary antibodies are listed in Table 3.

### 2.12. Determination of Lipid Peroxidation

Lipid peroxidation was evaluated by determining the production rate of thiobarbituric acid-reactive substrate using the thiobarbituric acid-reactive substances (TBARS) assay as described in [61]. The 4T1 cells were seeded in T75 flasks and exposed to different concentrations of IPA or Mito-TEMPO mitochondria-targeted antioxidant (5 μM)/GSH and NAC antioxidants (5 mM)/AHR (10 μM) and PXR (25 μM) inhibitors together with IPA (0.4 μM or 0.8 μM) for 24 h. Cells were washed in PBS and scraped; then, they were collected by centrifugation. After adding 8.1% SDS, 20% acetic acid, 0.8% thiobarbituric acid (TBA), and distilled water to the pellet, the samples were heated at 96 °C for 1 h. Samples were cooled down on ice and centrifuged. The absorbance of the supernatants was identified at 540 nm. The levels of 4-hydroxynonenal (4HNE)-modified proteins, as a marker for lipid peroxidation were also assessed using Western blotting.

### 2.13. Animal Study

Animal experiments were approved by the Institutional Animal Care and Use Committee at the University of Debrecen and the National Board for Animal Experimentation (1/2015/DEMÁB) and were performed according to the NIH guidelines (Guide for the care and use of laboratory animals) and applicable national laws. Animal studies are reported in compliance with the ARRIVE guidelines.

Experimental animals were BALB/c female mice (14–16 weeks of age, 20–25 g). Animals were randomized for all experiments. Mice were bred in the “specific pathogen-free” zone of the Animal Facility at the University of Debrecen and kept in the “minimal disease” zone during the experiments. No more than 5 mice were housed in each cage (standard block shape 365 × 207 × 140 mm, surface 530 cm^2^; 1284 L Eurostandard Type II. L from Techniplast). Cages were changed once a week on the same day. Animals had paper tubes to enrich their environment. The dark/light cycle was 12 h, and temperature was 22 ± 1 °C. Mice had ad libitum access to food and water (sterilized tap water). The animal facility was overseen by a veterinarian. A total of 20 female mice were used in the study: 10 randomly selected control and 10 IPA-fed mice.

4T1 cells were suspended (2 × 10^6^/mL) in ice-cold PBS-Matrigel (1:1, Sigma-Aldrich) at a 1:1 ratio. Twenty female BALB/c mice received 50 µL injections to their second inguinal fat pads on both sides (10^5^ cells/injection site). Tumor growth and animal wellbeing was controlled daily.

IPA treatment was administered by oral gavage at the dose of 1 nmol/g (0.18921 mg/kg) body weight once a day on each day of the experiment. The dose was planned not to exceed the serum reference concentration of IPA [40,49,50]. Animals received single daily oral IPA treatment. IPA stock was prepared in sterilized tap water at 100 × concentration (15 mM) for storage at −20 °C. IPA stock was diluted each day to a working concentration of 150 µM in sterile tap water before treatment. Animals received a daily oral dose of 100 µL/30 g bodyweight from the IPA solution (10 mice) or vehicle (sterilized tap water, 10 mice). Researchers involved in IPA and vehicle solution administration were blinded. Treatment was administered every day at the same time between 9:00 and 11:00. Animals were sacrificed on day 14 post grafting by cervical dislocation; then, primary tumors and metastases were collected for subsequent analysis.

Upon autopsy, primary tumors were visually evaluated and scored based on their infiltration rate into surrounding tissues and the macroscopic appearance of the tumor [27,28]. Tumors were classified as a “low infiltration” class if the primary tumor remained in the mammary fat pads without any attachment to muscle. A “medium infiltration” tumor means that the tumor mass attached to the muscle tissue, but it did not penetrate to the abdominal wall. In case the tumor grew into the muscle tissue and totally penetrated the abdominal wall, it was scored as a “high infiltration” tumor. For the assessment of primary tumor infiltration rate, the researchers were blinded. Both primary and metastatic tumor masses were removed from the animals and were measured on analytical balance in preweighed Eppendorf tubes. From the sections of HE-stained, formalin-fixed, paraffin-embedded tumor tissues tumor-infiltrating lymphocyte (TIL) content was determined as the number of TILs per 100 tumor cells.

### 2.14. Human Studies

We assessed the abundance of the bacterial TnaA coding DNA in human fecal DNA samples. We used three cohorts in the study.

#### 2.14.1. Cohort 1

Cohort 1 consisted of fecal DNA samples and were used for the quantitation of the TnaA coding DNA. The cohort involved 48 controls and 46 breast cancer patients.

The human feces samples were collected from healthy volunteers and breast cancer patients by collaborators at the National Cancer Institute (NCI), Kaiser Permanente Colorado (KPCO), the Institute for Genome Sciences at the University of Maryland School of Medicine, and RTI International. The study protocol and all study materials were confirmed by the Institutional Review Boards at KPCO, NCI, and RTI International (IRB number 11CN235). The study results were published in [3]. Informed consent was obtained from the study participants. That cohort was designed and powered to compare healthy controls versus breast cancer patients, but we also stratified patients using the available clinical data.

#### 2.14.2. Cohort 2

This cohort was used in assessing the intratumoral expression of IPA receptors. The tissue microarray (TMA) study was carried out using archived tissue blocks of 88 breast cancer patients.

Formalin-fixed, paraffin-embedded tissues from breast cancer surgeries were collected, and tissue microarray (TMA) blocks were built [62]. The cohort is described in [30]. The study was approved by the local ethical committee at the University of Debrecen. That cohort was designed and powered to compare healthy controls versus breast cancer patients, but we also stratified patients using the available clinical data.

#### 2.14.3. Cohort 3

Fecal protein expression was assessed in a cohort of female breast cancer patients of 35 participants with a mean age of 57 years. Samples from stage I–III and Nottingham grade 1–3 patients were used in the study. The average stage and grade were not statistically different amongst the groups. The cohort was published in [57].

The collection and biobanking of feces were authorized by the Hungarian national authority (ETT). Patients and healthy volunteers meeting the following criteria were excluded from the study: (1) has a previous history of breast cancer or had been operated due to neoplasia, (2) has a disease of unknown origin, (3) has a chronic contagious disease, (4) had contagious diarrhea 6 months prior to enrollment, (5) taken antibiotics within the 6 months prior to enrollment, (6) had chemotherapy, biological therapy, or immunosuppressive therapy 6 months prior to enrollment, (7) used intravenous drugs 12 months prior to enrollment, (8) had piercing, tattooing, acupuncture, or other endangering behavior or action 12 months prior to enrollment, (9) exposure to an allergen to which the enrolled individual had been sensitized to, or (10) underwent colonoscopy 12 months prior to enrollment. The first morning feces was sampled; samples were frozen and deposited in the biobank within two hours after defecation. Samples were stored at −70 °C until subsequent use. We obtained informed consent from study participants. The patient’s routine pathological data were assessed in the study and were compared to the fecal expression of *E. coli* TnaA.

### 2.15. Tissue Microarray, Immunohistochemistry and Analysis

TMA in combination with immunohistochemistry was performed as in [62]. From every block, there were three replicates, and the staining was assessed applying an H-score system [63]. In immunohistochemical studies, Leica Bond Max^TM^ protocol was used. The antibodies and conditions are listed in Table 4.

### 2.16. Database Screening

The kmplot.com database [64] was used to examine the connection between gene expression levels (AHR and PXR) and breast cancer survival in humans. The case numbers are listed in Table 5. 

The sequence of the *TnaA* ORFs was retrieved from the KEGG (www.genome.jp/kegg/), the PATRIC (www.patricbrc.org/) and the Uniprot (www.uniprot.org) databases

### 2.17. Statistical Analysis

For the comparison of two groups, we used the two-tailed Student’s *t*-test, unless stated otherwise. Fold data were log_2_ transformed to achieve normal distribution. Statistical significance was determined for multiple comparisons with one-way analysis of variance test (ANOVA) followed by Tukey’s or Dunnett’s honest significance difference (HSD) post-hoc test. All data are presented as average ± SEM unless stated otherwise. Texas Red-X Phalloidin-labeled fluorescent pictures were analyzed using Cell Profiler 2.0 followed by Advanced Cell Classifier 3.0. FACS results were analyzed using Flowing Software 2.0. Statistical analysis was done using GraphPad Prism 7 software unless stated otherwise.

## 3. Results

### 3.1. Indolepropionic Acid Reduces the Progression of Breast Cancer In Vivo

**Figure 1:**
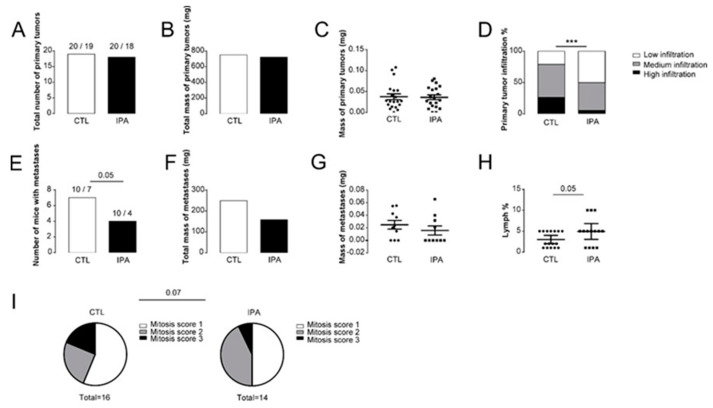
Supplementation of mice transplanted with 4T1 breast cancer cells with indolepropionic acid (IPA) reduces metastatic burden. Female Balb/c mice (*n* = 10/10, 3 months of age) were grafted with 4T1 cells and were treated with IPA (1 nmol/g q.d. p.o.) or vehicle (VEH) (*n* = 10/10) for 14 days; then, the mice were terminated. Upon autopsy (**A**), the total number and (**B**) total and (**C**) individual mass of primary tumors were determined. (**D**) The infiltration of the primary tumors to the surrounding tissues was scored. The (**E**) number of mice with metastasis were plotted, and the (**F**) total, as well as the (**G**) individual mass of the metastases were measured. In tissue sections of formalin-fixed, paraffin-embedded tissue specimens from the primary tumors (**H**), lymphocyte infiltration and (**I**) mitosis score were determined. Statistical significance was determined using Student’s *t*-test, except for panels D, E, and I, where chi-square tests were conducted. *** indicate statistically significant difference between vehicle and treated groups at *p* < 0.001.

As a first step, we examined the effects of IPA supplementation on tumor growth by grafting 4T1 breast cancer cells to 20 female Balb/c mice to the left and right side (200,000 cells to each site). Half of the mice received vehicle (sterilized tap water) as control, while the other half of the mice received IPA (1 nmol/g bodyweight). *Per os* IPA treatment did not inhibit the growth of the primary tumor, as neither the number of tumors, nor the total, nor the mass of primary tumors differed with IPA treatment (Figure 1A–C). However, IPA treatment drastically reduced the level of infiltration to the surrounding tissues (Figure 1D). IPA treatment reduced the number of mice bearing metastases (Figure 1E) and the total mass of metastases (Figure 1F), although the mass of the individual metastases did not change (Figure 1G). We assessed the histology of the primary tumors and found that the infiltration of lymphocytes was increased and the mitosis score of the tumor cells was decreased in the IPA-treated mice (Figure 1H).

### 3.2. Indolepropionic Acid Inhibits Hallmarks of Cancer

**Figure 2:**
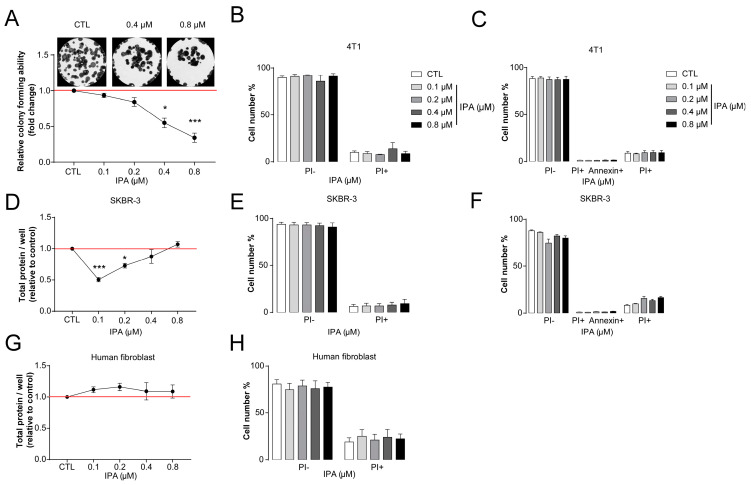
Indolepropionic acid (IPA) reduces cell proliferation in cellular models of breast cancer. (**A**) 500 cells/well 4T1 cells were seeded in 6-well plates and then treated with IPA in the concentrations indicated for 7 days. Then, colonies were stained according to May–Grünwald–Giemsa and analyzed using the ImageJ software (*n* = 3). (**B**) 4T1 cells were seeded in 6-well plates (75,000 cells/well) and treated with the indicated concentrations of IPA for 24 h and stained with propidium-iodide; then, they were analyzed by flow cytometry. (**C**) 4T1 cells (75,000 cells/well in 6-well plates) were treated with IPA in the concentrations indicated for 24 h; the ratio of necrotic and apoptotic cells were determined with staining by propidium–iodide–FITC Annexin double staining using the V/Dead Cell Apoptosis Kit and measured by flow cytometry (*n* = 3). (**D**) SKBR-3 (5000 cells/well) were seeded in 96-well plates and then were treated with IPA in the concentrations indicated for 24 h; then, total protein content was assessed in sulforhodamine B (SRB) assays (*n* = 3). (**E**) SKBR-3 (200,000 cells/well) cells were seeded in 6-well plates and were treated with IPA in the concentrations indicated for 24 h. The ratio of necrotic and apoptotic cells were determined with staining by propidium–iodide; then, they were analyzed by flow cytometry. (**F**) SKBR-3 (200,000 cells/well) cells were seeded in 6-well plates and were treated with IPA in the concentrations indicated for 24 h. The ratio of necrotic and apoptotic cells were determined with staining by propidium–iodide–FITC Annexin double staining using the V/Dead Cell Apoptosis Kit and measured by flow cytometry (*n* = 3). (**G**) Human fibroblast (7500 cells/well) cells were seeded in 96-well plates and then were treated with IPA in the concentrations indicated for 24 h; then, the total protein content was assessed in SRB assays (*n* = 3). (**H**) Human fibroblast (20,000 cells/well) cells were seeded in 6-well plates and then were treated with IPA in the concentrations indicated for 24 h. Then, the ratio of necrotic and apoptotic cells were determined with staining by propidium–iodide and analyzed by flow cytometry (*n* = 3). Fold data were log2 transformed to achieve normal distribution. Statistical significance was determined using ANOVA test followed by Tukey’s post-hoc test, except for panels D, E, and F, where Dunnett’s post-hoc tests were conducted. * or *** indicate statistically significant difference between control and treated samples at *p* < 0.05 or *p* < 0.001, respectively.

IPA reduced the proliferation of 4T1 cells in colony-forming assays (Figure 2A).

Other bacterial metabolites that possess cytostatic effects in breast cancer, such as lithocholic acid or cadaverine are only cytostatic; they are not toxic [27,28]. Therefore, we assessed IPA in concentrations corresponding to the reference concentration. In good agreement with the previously identified metabolites, IPA did not increase either the proportions of propidium–iodide positive necrotic or the Annexin–FITC–propidium–iodide double-positive apoptotic cells (Figure 2B,C).

We also assessed whether the effects, described above, are specific for only 4T1 cells, but they can also be elicited in another breast cancer cell line. IPA, in a similar concentration range used for 4T1 cells, decreased cell proliferation as measured in SRB assays; it did not increase necrotic or apoptotic cell death in SKBR-3 (Figure 2D–F).

Finally, we found that IPA does not inhibit cell proliferation or exert cytotoxicity in primary, non-transformed human fibroblasts (Figure 2G,H), similarly to other cytostatic metabolites [27,28,65].

**Figure 3:**
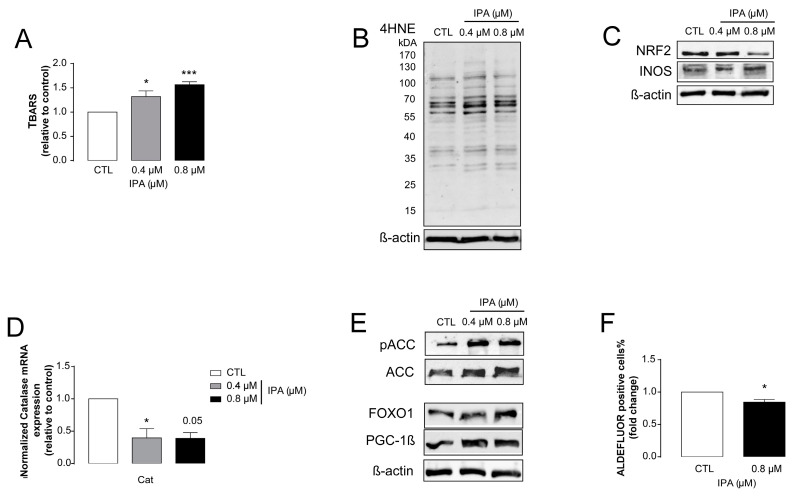
Indolepropionic acid (IPA) induced oxidative stress, cellular energy stress, and decreased the proportions of cancer stem cells. 500,000 cells/well 4T1 cells were treated with IPA in the concentrations indicated for 24 h; then, (**A**) lipid peroxidation was measured by TBARS assay, and (**B**) 4HNE expression was assessed by Western blotting (representative figure, *n* = 3). In the same cells (**C**), the protein expression of NRF2 (at 68 kDa) and iNOS were determined by Western blotting (*n* = 3), while (**D**) the mRNA expression of catalase (cat) was determined by RT-qPCR (*n* = 3). (**E**) The expression of the indicated proteins (pACC, ACC, FOXO1, and PGC-1*β*) were determined by Western blotting (*n* = 3, except for PGC-1*β*, where *n* = 2). (**F**) 100,000 cells/well 4T1 cells were treated with the indicated concentration of IPA for 24 h; then, the proportions of aldehyde dehydrogenase-positive cells were determined in Aldefluor assays using flow cytometry (*n* = 3). For Western blots, a typical experiment was displayed. Fold data were log2 transformed to achieve normal distribution. Statistical significance was determined using the ANOVA test followed by Dunnett’s post-hoc test, except for panel F, where Student’s *t*-test was used. * and *** indicate statistically significant difference between control and treated samples at *p* < 0.05 and *p* < 0.001, respectively.

Next, we assessed whether IPA can modulate other cancer hallmarks that were regulated by other bacterial metabolites [34]. Although the role of oxidative stress in breast cancer was long debated, recent studies consistently showed that increased oxidative stress is responsible for cytostasis or cancer cell death [30,66,67,68,69,70]. We assessed different oxidative stress markers to explore the redox status of IPA-treated cancer cells. IPA treatment increased lipid peroxidation, as measured by TBARS assay and by the formation of 4HNE (Figure 3A,B), which is indicative of enhanced oxidative stress. Enhanced oxidative stress was in parallel with the reduction in the expression of nuclear factor erythroid 2-related factor 2 (NRF2), which is a transcription factor responsible for the expression of antioxidant enzymes, such as catalase. In parallel, we observed the enhanced expression of inducible nitric oxide synthase (iNOS), an enzyme promoting nitrosative stress (Figure 3C,D).

Finally, we assessed markers of cellular energy stress (pACC, ACC, FOXO1, and PGC1β) and cancer stem cell-ness (aldehyde dehydrogenase [71]). IPA treatment reduced the proportions of cancer stem cells (Figure 3F), while inducing the markers of cellular energy stress (Figure 3E).

**Figure 4:**
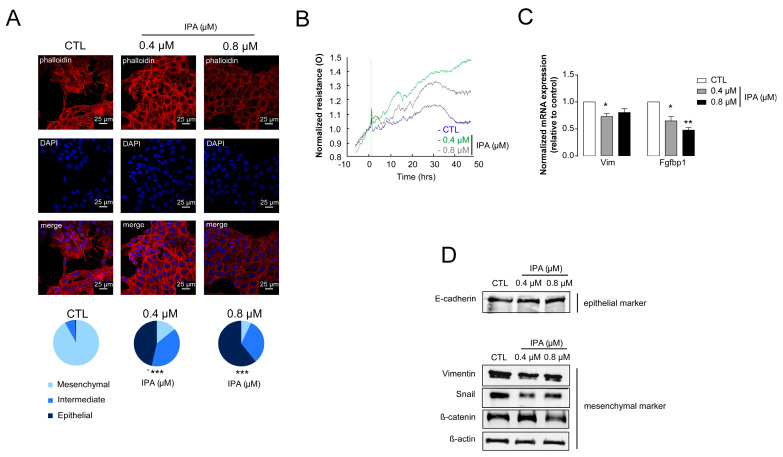
Indolepropionic acid (IPA) induced mesenchymal-to-epithelial transition (EMT). 100,000 cells/well 4T1 cells were treated with IPA in the concentrations indicated for 24 h; then, (**A**) cellular morphology was observed using Texas Red-X Phalloidin and DAPI staining (representative figure, *n* = 3). Scale bar corresponds to 25 μm. Mesenchymal cells are characterized by stress filaments that are absent in epithelial cells; for details on morphology, see [27] and Figure S3. (**B**) Normalized resistance was measured in electric cell–substrate impedance sensing (ECIS) impedance-based experiments (representative figure, mean ± SD, *n* = 1). (**C**,**D**) In IPA-treated 4T1 cells, the expression of the indicated genes were determined in (**C**) RT-qPCR *(n* = 3) and (**D**) *Western blotting* (*n* = 3). *β*-actin was used as a loading control. For Western blots, a typical experiment was displayed. Fold data were log2 transformed to achieve normal distribution. A statistical significance was determined using the ANOVA test followed by Dunnett’s post-hoc test, except for panel D, where a chi-square test were conducted. *, ** and *** indicate statistically significant difference between control and treated samples at *p* < 0.05, *p* < 0.01 or *p* < 0.001, respectively. Abbreviations: Vimentin (Vim), fibroblast growth factor-binding protein1 (FgfBp1), snail family transcriptional repressor-1 (Snail) and β-catenin) E-cadherin, zonula occludens-1 (ZO1).

Another important feature of breast cancer is epithelial-to-mesenchymal transition (EMT). The IPA treatment of 4T1 cells reverted EMT that was evidenced by the dose-dependent conversion of 4T1 cells to epithelial morphology (Figure 4A).

In good agreement with that, IPA treatment induced the resistance of a cellular monolayer to the electric current, indicative of better cell-to-cell and cell-to-surface binding (Figure 4B). Finally, we assessed epithelial and mesenchymal markers at the protein and mRNA level. IPA treatment induced the expression of mesenchymal makers (Vimentin (Vim), fibroblast growth factor-binding protein1 (FgfBp1), snail family transcriptional repressor-1 (Snail), and β-catenin; and it upregulated the expression of E-cadherin, which is an epithelial marker (Figure 4C,D).

### 3.3. IPA-Induced Cytostatic and Antineoplastic Effects Are Mediated by Reactive Species Production

**Figure 5:**
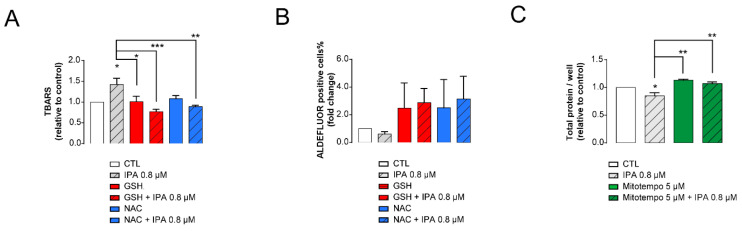
Indolepropionic acid (IPA)-elicited oxidative stress has central role in mediating IPA-elicited antineoplastic effects. 4T1 cells (500,000 cells/well, TBARS; 100,000 cells/well, ALDH; 1500 cells/well, SRB) were treated with IPA in the concentrations indicated for 24 h with or without the antioxidants, as indicated. Subsequently, (**A**) thiobarbituric acid-reactive substances, (**B**) the fraction of Aldefluor-positive cells and (**C**) total protein content was determined. Fold data were log2 transformed to achieve normal distribution. Statistical significance was determined using the ANOVA test followed by Dunnett’s post-hoc test. *, **, and *** indicate statistically significant difference between control and treated samples at *p* < 0.05, *p* < 0.01 or *p* < 0.01, respectively. Abbreviations: TBARS—Thiobarbituric acid-reactive substances, ALDH—Aldehyde dehydrogenase, SRB—Sulforhodamine B, GSH—reduced glutathione, NAC—N-acetyl-cysteine.

EMT, as well as cancer stem cell characteristics are modulated by reactive species [72,73]. With enhanced oxidative stress upon IPA treatment, it was likely that the suppression of these features may be oxidative stress-driven. To get an insight on whether IPA can modify oxidative stress to exert cytostatic effects on breast cancer cells, we assessed the effects of thiol reductants glutathione (GSH) and *n*-acetyl-cysteine (NAC), as well as mitochondria-targeted antioxidant Mito-TEMPO, on IPA-mediated cancer hallmarks. As expected, the application of general thiol reductants (reduced glutathione (GSH) and N-acetyl-cysteine (NAC)) reduced the IPA-induced increase in thiobarbituric acid-reactive substances (Figure 5A), suggesting a reduction in IPA-induced oxidative stress.

Furthermore, GSH and NAC treatment protected against the IPA-induced decrease in ALDH1-positive cancer stem cells (Figure 5B). More interestingly, a mitochondrial antioxidant, Mito-TEMPO, protected against IPA-induced cytostasis (Figure 5C). These data suggest that IPA-induced reactive species production has a central role in eliciting the widespread antineoplastic effects of IPA.

### 3.4. IPA Exerts Its Effects through Aryl-Hydrocarbon Peceptor (AHR) and Pregnane X Receptor (PXR) 

**Figure 6:**
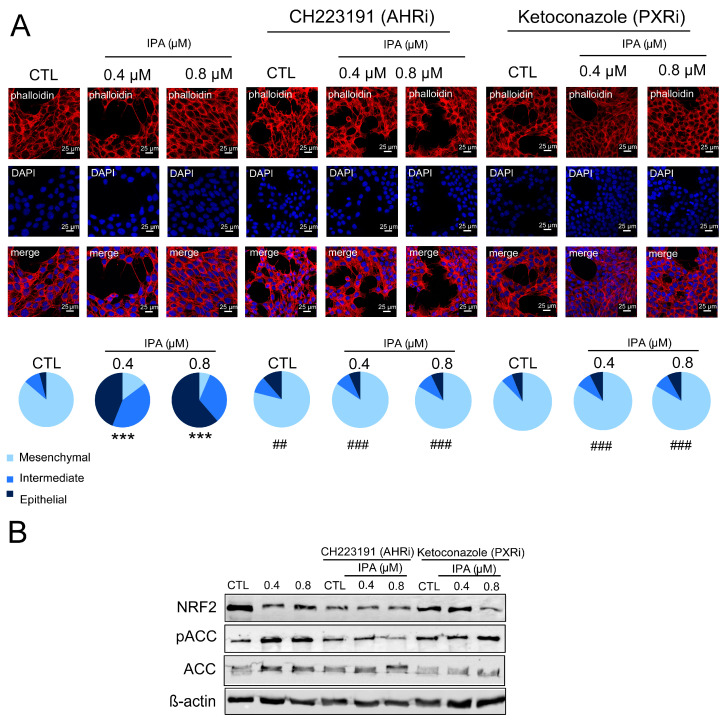
AHR and PXR are responsible for the IPA-elicited antineoplastic effects. 100,000 cells/well 4T1 cells were treated with IPA in the concentrations indicated for 24 h with or without the inhibitors, as indicated. Subsequently, (**A**) the actin cytoskeleton was stained using phallodin-Texas Red, and morphology was assessed using confocal microscopy. (**B**) On the same cells, the expression of the indicated proteins were determined by Western blotting. Fold data were log2 transformed to achieve normal distribution. Statistical significance was determined using an ANOVA test followed by Dunnett’s post-hoc test, except for panel A, where a chi-square test was conducted. *** indicate statistically significant difference between control and IPA treated samples at *p* < 0.01. ## and ### indicate significant difference between control and CH223191 or ketoconazole-treated samples at *p* < 0.01 or *p* < 0.001, respectively. Abbreviations: PXRi—PXR inhibitor, AHRi—AHR inhibitor, ACC—acyl-CoA carboxylase (an AMPK target protein).

IPA has multiple receptors [40], of which we investigated pregnane X receptor (PXR) and aryl hydrocarbon receptor (AHR) in detail in our study. We used pharmacological inhibitors of AHR (CH223191) and PXR (ketoconazole [51,52]) to interrogate the involvement of IPA receptors. The inhibition of AHR and PXR abrogated or quenched several IPA-induced features, namely, the IPA-induced reversion of EMT (Figure 6A), and the induction of NRF2 and AMPK (as marked by the phosphorylation of acyl-CoA carboxylase, or ACC) (Figure 6B).

**Figure 7:**
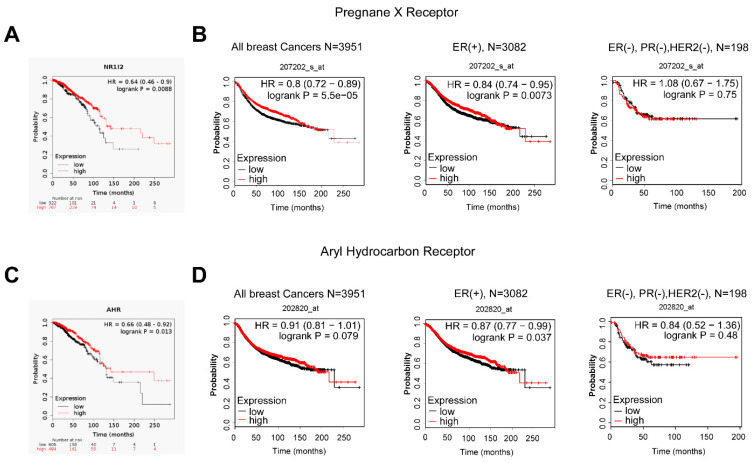
Higher expression of pregnane X receptor and aryl hydrocarbon receptor prolongs survival in breast cancer. The effect of expression of (**A**) AHR or (**B**) PXR on survival in breast cancer was analyzed by kmplot.com, which is a freely accessible database. On panel (**A**), the effect of PXR expression on survival is depicted with the data acquired from RNAseq experiments. On panel (**B**), the effect of PXR expression on survival is depicted with the data acquired from microarray experiments, and patients were stratified as a function of receptor expression. On panel (**C**), the effect of AHR expression on survival is depicted with the data acquired from RNAseq experiments. On panel (**D**), the effect of AHR expression on survival is depicted with the data acquired from microarray experiments, and patients were stratified as a function of receptor expression. Total survival rates were assessed, and all samples are represented. Abbreviations: NR1I2—pregnane X receptor, Aryl hydrocarbon receptor (AHR), Pregnane X receptor (PXR) The database was assessed the 19th February 2020.

We assessed whether the expression of AHR and PXR correlates with survival in human breast cancer using the kmplot.com database [64]. First, we assessed the RNAseq datasets and found that the quartile of the patient population with the highest expression has advantage in survival over the quartile with the lowest expression both in the case of PXR (Figure 7A) and AHR (Figure 7C).

Next, we assessed the microarray data as well, where we were able to stratify patients as a function of receptor expression (Figure 7B,D). The expression level of AHR did not have a clear impact on breast cancer survival; nevertheless, there was a trend for better survival in the high expression quartile, similarly to the RNAseq data (Figure 7D). Importantly, a higher expression of PXR provided better survival with breast cancer, particularly during the first 4–12 years after diagnosis and for estrogen receptor-positive cancer cases (Figure 7B, Table 5).

**Figure 8:**
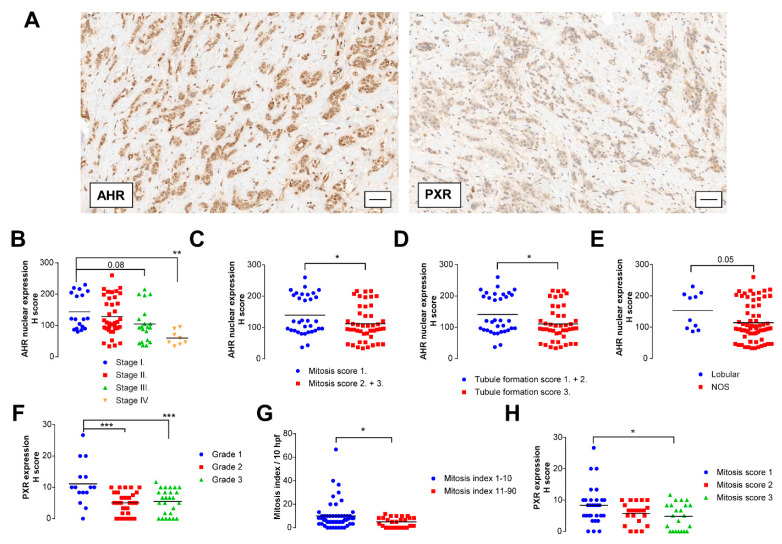
Higher intratumoral expression of pregnane X receptor (PXR) and nuclear aryl hydrocarbon receptor (AHR) shows correlation with low-grade and lower-mitosis breast cancers. (**A**) TMA was stained with the indicated antibodies. A typical staining pattern is shown. The bar is equivalent of 50 µm. Cases in TMA were scored for receptor expression using the H-score system. (**B**–**E**) H-score of the nuclear expression of the AHR receptor were related to (**B**) stage of the disease, (**C**) mitosis score, (**D**) tubule formation, and (**E**) histological subtype. (**F**–**H**) H-score of the expression of PXR receptor were related to (**F**) grade of the disease, (**G**) mitosis score, and (**H**) mitotic index. Statistical significance on panels B and F was determined using an ANOVA test followed by Dunnett’s post-hoc test, while on panels C, D, E, G, and H, Student’s *t*-test was used. Stage 0 (in situ carcinoma) and stage 1 patients, Mitosis score 2 and 3 patients, and tubule formation score 1 and 2 patients were handled together due to the low number of cases. Abbreviations: Aryl hydrocarbon receptor (AHR), Pregnane X receptor (PXR), no special type (NOS). *, ** and *** indicate statistically significant difference between control and treated samples at *p* < 0.05, *p* < 0.01 or *p* < 0.001, respectively.

These in silico data were complemented by characterizing the intratumoral expression of AHR and PXR (Figure 8A) in a tissue microarray (TMA) consisting of 88 patients (the TMA was published in [30], Cohort 2).

When AHR expression was considered in the whole cell, it did not show any consequent change (data not shown); nevertheless, when we scored only the nuclear fraction of AHR that represents the active AHR [74], we found that the AHR nuclear score (i.e., AHR activity) decreased with the progression of the disease (Figure 8B). Furthermore, AHR activity was lower in highly proliferating tumors (Figure 8C), in tumors with lower differentiation (Figure 8D), and in not otherwise specified (NOS) tumors as compared to lobular tumors (Figure 8E). PXR expression decreased as the Nottingham grade of the patients increased (Figure 8F) and in tumors with a high proliferation rate (Figure 8G,H).

### 3.5. Microbial Indole Biosynthesis by the Gut Microbiome Is Repressed in Early Stages of Breast Cancer

To assess the link between bacterial IPA biosynthesis and breast cancer behavior, we assessed the capability of the gut microbiota to synthesize indole derivatives. To that end, we identified bacterial species in which tryptophanase operon was mapped using a database search and designed primers to the regions coding for TnaA. We measured the abundance of the DNA coding for TnaA in fecal DNA of recently diagnosed patients versus healthy controls (published in [3], Cohort 1) using qPCR.

**Figure 9:**
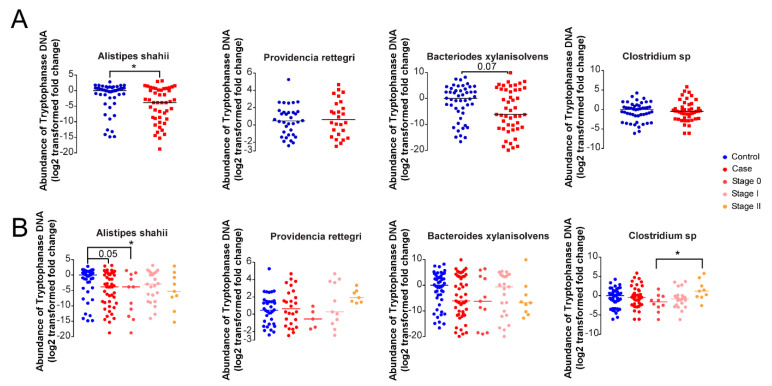
The fecal expression of TnaA shows correlation with the pathological and clinical features of breast cancer. (**A**,**B**) The abundance of bacterial TnaA DNA was assessed in human fecal DNA samples from cohort study. The c_t_ values lower than 45 are shown in *Providencia rettgeri*. Median values indicated by a line. On panel A, all patients and controls are compared, statistical significance was calculated using Student’s *t*-test. On panel B, patients were stratified based on the stage of the disease, and statistical comparison was made using an ANOVA test followed by Dunnett’s or Tukey’s post-hoc tests. Fold data were log2 transformed to achieve normal distribution. * indicate a statistically significant difference between control and treated samples at *p* < 0.05.

When comparing patients against healthy individuals, the abundance of TnaA in *Providencia rettgeri* and *Alistipes shahii* was significantly reduced in patients, and there was a similar trend in *Bacteroides xylanisolvens* (Figure 9A). Next, we stratified patients as a function of the stage of their disease. The decrease in TnaA abundance was accentuated in stage 0 (in situ carcinoma) patients not only for P. *rettgeri*, *A. shahii*, and *B. xylanisolvens*, but also for a general probe on *Clostridium* species (Figure 9B).

### 3.6. Fecal Protein Expression of *E. coli* TnaA Correlates with the Number of Tumor Infiltrating Lymphocytes

**Figure 10:**
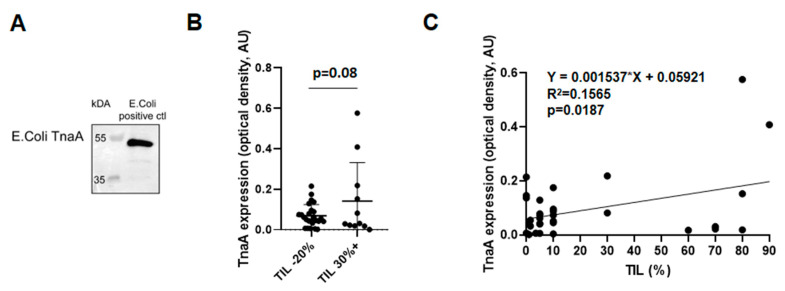
The fecal protein expression of *E. coli* TnaA is higher in patients with higher proportions of tumor-infiltrating lymphocytes. (**A**) *E. coli* total lysate was run on an SDS-PAGE gel, transferred to nitrocellulose membrane, and probed with an anti-TnaA antibody. (**B**,**C**) Fecal samples of 36 low tumor-infiltrating lymphocyte (TIL) patients (0–20% TIL) and 11 of high TIL patients (30%< TIL) were assessed by Western blotting using an anti-TnaA antibody. Protein content-normalized values were obtained. Values were tested for outliers using Grubb’s method; one value was omitted from the low TIL group. (**B**) Normalized TnaA expression was plotted. (**C**) On values from low and high TIL patients, liner regression was performed.

We wanted to enlarge the scope of our findings by assessing protein-level changes to bacterial indol biosynthetic enzymes. The antibody commercially available was against the TnaA protein of *E. coli* origin (Figure 10A).

We determined the *E. coli* TnaA expression in the feces of breast cancer patients from Cohort 3. We stratified the patients based on the number of tumor-infiltrating lymphocytes (TIL) and found that there was a trend of higher TnaA expression in patients with higher TIL (Figure 10B). In good agreement with that finding, we found correlation between TIL and *E. coli* TnaA expression in breast cancer patients by performing linear regression (Figure 10C).

## 4. Discussion

**Figure 11:**
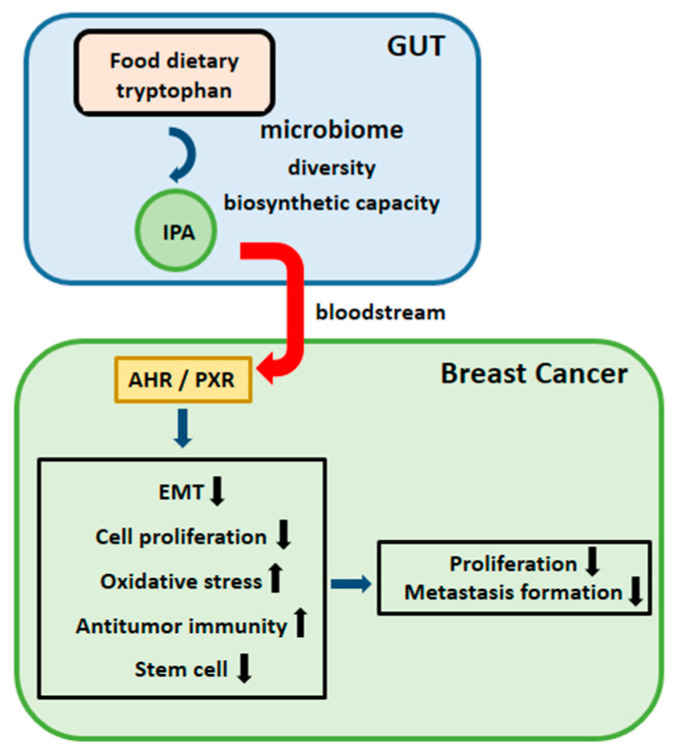
Schematic representation of IPA-elicited effects to breast cancer cells.

In this study, we found that a bacterial metabolite, indolepropionic acid (IPA), has antineoplastic features (Figure 11).

IPA supplementation decreased the infiltration of the primary tumor to surrounding tissues, the number of metastases, cellular movement, and diapedesis, while at the same time, it induced antitumor immune response, mesenchymal-to-epithelial transition, oxidative stress, and influenced metabolism through two IPA receptors, AHR and PXR (both are steroid and xenobiotic receptors [75,76,77]). Similar to the effects of other antineoplastic metabolites, such as lithocholic acid or cadaverine, IPA did not exert its cytostatic effects on non-transformed cells [27,28,65], exhibiting tumor cell-specific effects. Finally, we provided evidence that IPA exerts its antineoplastic modulation through the AHR and particularly the PXR receptors.

Although the reports on the oncobiome in breast cancer are conflicting, the majority of the reports show suppressed diversity [1,2,3,4,5,8,9,34,78,79,80,81] and as a consequence, limited biosynthetic capacity [4], which is probably translated to a reduced availability of bacterial metabolites in the serum and in the tumor [82]. It is of note that common changes to the microbiome among the different studies were identified [12]. Furthermore, the breast’s own microbiome also changes in breast cancer patients, but the contribution of that local microbiome depot to breast cancer is unknown [83,84,85,86,87,88]. 

A diverse eubiome is required for normal antitumor immunity [38,89,90]. In addition to that, bacterial metabolites, among them, tryptophan and its metabolites [46], are capable of modulating immunity [27,77]. Tryptophan and its downstream metabolites, including indoles, exert their effects through the AHR and PXR receptors [91,92,93]. A dietary restriction of tryptophan that subsequently limits indole production has immunosuppressive effects in an AHR-dependent fashion [94], which correlates well with the antitumor immune response we observed in our study. High TnaA expression is indicative of higher IPA production, and it is directly proportional with the number of tumor-infiltrating lymphocytes. It is also important to note that tryptophan (metabolite)-dependent modulation of the immune system can shape the composition of the microbiome [95]. IPA treatment increased oxidative and nitrosative stress through the inhibition of the expression of NRF2 and a subsequent reduction in cellular antioxidant defense (e.g., downregulation of caspase expression).

In addition, IPA induced iNOS expression and enhanced mitochondrial reactive species production. NRF2 downregulation and iNOS overexpression was shown for lithocholic acid and indoxylsulfate, which are other cytostatic bacterial metabolites, although neither of these metabolites induced mitochondrial reactive species production [30,96]. NRF2 and iNOS have profound roles in setting the redox balance in cancer cells [67,97]. Increased oxidative and nitrosative stress is cytostatic in breast cancer [30,66,69,70]. An increase in reactive species production is vital to exert the cytostatic properties of IPA. Furthermore, oxidative stress is a key regulator of cancer cell stem-ness [98], and higher levels of oxidants switch cancer stem cells to lose their stem properties [99,100,101]. IPA treatment reduced the proportions of cancer stem cells, which is an effect that was reverted by the addition of thiol reductants. The oncobiome has a pivotal role in regulating EMT and metastasis formation [27,28,29,31,32,96]. 

IPA induced mesenchymal-to-epithelial transition (MET), as found with other microbial metabolites [27,28,96,102]. Inducing MET slows down cell movement, diapedesis, and metastasis formation. In our models, we observed that IPA supplementation reduced metastasis formation.

In addition to these findings, we showed that IPA induces AMPK, FOXO1, and PGC1β, which are enzymes inducing mitochondrial biogenesis, in an AHR/PXR-dependent fashion. The activation and overexpression of these enzymes were shown to be associated with better survival in breast cancer [27,53,103,104].

Are these observations relevant to human breast cancer? Tryptophan and indole metabolism is tightly associated with breast cancer and breast cancer survival. High extracellular tryptophan levels associate with worse survival in breast cancer (Table S8 [105]). The levels of an indole derivative, 3-indoxyl-sulfate—a downstream metabolite of tryptophan degradation and indole propionic acid formation—is downregulated both in estrogen receptor-positive and negative cases ([106] Additional file 3, Table S3 Line 44). Furthermore, in breast tumors, there is a negative correlation between Ki67 positivity (a proliferation marker) and 3-indoxyl-sulfate levels ([106] Additional file 9, Table S8 line 130). These data suggest that indole derivatives support the survival of patients with breast cancer and that their levels are downregulated with progressive disease. In addition to these, we showed that the IPA biosynthetic capacity of the microbiome is reduced in women newly diagnosed with breast cancer, especially for women with in situ carcinoma, mirroring associations with other gut microbial metabolites, lithocholic acid [27] and cadaverine [28]. These observations are also in good correlation with the intratumoral expression pattern and activity of AHR and PXR that are downregulated with the progression of the disease, or were lower in aggressive, highly proliferative, and undifferentiated breast cancer cases. The model that we can build from the data we represented and from the available literature is that in breast cancer, oncobiosis bacterial tryptophan metabolism is suppressed, and this seems to be the most profound in early stage, in situ carcinoma cases. Decreases in IPA release the cytostatic lockdown of breast cancer cells. Later stage and aggressive cases are characterized by a lower expression or lower activity of metabolite-elicited signaling. Apparently, oncobiosis seems to play a role in breast carcinoma progression but not in the initiation of the disease.

As we noted above, accumulating evidence suggests a functional role of the oncobiome in breast cancer, as seen in human population-based studies and animal studies on antibiotic use, metabolite supplementation studies, nutritional studies [107], and pharmacological studies [108] such as the one we are presenting hereby. These studies open new approaches in breast cancer treatment through identifying bacterial metabolites and elements of the oncobiome that can be exploited in therapy.

## 5. Conclusions

In this paper we showed that IPA has cytostatic and antineoplastic properties in breast cancer. IPA reduced the proportions of cancer stem cells and the proliferation, movement, and metastasis formation of cancer cells. These were achieved through inhibiting epithelial-to-mesenchymal transition, inducing oxidative and nitrosative stress, and boosting antitumor immune response. IPA exerted its effects through aryl hydrocarbon receptor (AHR) and pregnane X receptor (PXR) receptors. A higher expression of PXR and AHR supported better survival in human breast cancer patients, highlighting the importance of IPA-elicited pathways in cytostasis in breast cancer. Furthermore, AHR activation and PXR expression related inversely to cancer cell proliferation level and to the stage and grade of the tumor. The fecal microbiome’s capacity for IPA biosynthesis was suppressed in women newly diagnosed with breast cancer, especially with stage 0. Bacterial indole biosynthesis showed correlation with lymphocyte infiltration to tumors in humans. Bacterial metabolites (e.g., lithocholic acid, short chain fatty acids or cadaverine), among them, IPA, have a pivotal role in regulating the progression but not the initiation of the disease.

## Figures and Tables

**Table 1:** Murine and human primers used in reverse transcription-coupled PCR (RT-qPCR) reactions.

**Gene Symbol**	**Murine Forward Primer (5′–3′)**	**Murine Reverse Primer (5′–3′)**
CAT	CCTTCAAGTTGGTTAATGCAGA	CAAGTTTTTGATGCCCTGGT
VIM	CTCCAGAGAGAGGAAGCCGAAAG	CCTGGATCTCTTCATCGTGCAGT
FgfBp1	CAAGGTCCAAGAAGCTGTCTCCA	AGCTCCAAGATTCCCCACAGAAC
Cyclophilin A	TGGAGAGCACCAAGACAGACA	TGCCGGAGTCGACAATGAT
36B4	AGATTCGGGATATGCTGTTGG	AAAGCCTGGAAGAAGGAGGTC
**Gene Symbol**	**Human Forward Primer (5′–3′)**	**Human Reverse Primer (5′–3′)**
AHR	TTGAACCATCCCCATACCCCAC	GAGGTTCTGGCTGGCACTGATA
PXR	AGTGAAGGTTCCCGAGGACATG	TTGTCACAGAGCATACCCAGCA
Cyclophilin A	GTCTCCTTTGAGCTGTTTGCAGAC	CTTGCCACCAGTGCCATTATG
36B4	CCATTGAAATCCTGAGTGATGTG	GTCGAACACCTGCTGGATGAC

**Table 2:** Primers for the determination of abundance of tryptophanase (TnaA) using RT-qPCR reactions.

Gene Symbol	Forward Primer (5′–3′)	Reverse Primer (5′–3′)
*Alistipes shahii*	GCCTCGAAGACGATCAAGGAGAT	TCGAACATGATGCTGAACGACAT
*Providencia rettegri*	CGTTTACGTGAGGGAGGGATTTC	ACCGCACGAACACCAGATTCTAA
*Bacteriodes xylanisolvens*	AACTGGAAATCCCGTTCAAAGGA	GTACGGGTTTGCCGTATTTGTCA
*Clostridium sp.*	GATGGAACGTCCAAACACTTTCG	ATATTTTCCGCCTTCCGGAACTT

**Table 3:** List of antibodies used for Western blot.

Antibody	Dilution	Vendor
4-HNE	1:1000	Abcam (ab46545)
Nitrotyrosine	1:1000	Millipore (06–284)
iNOS	1:1000	Novus (NB300–605)
NRF2	1:1000	Abcam (ab31163)
LC3	1:1000	Cell Signaling (#13082)
Phospho-AMPKα (Thr172)	1:1000	Cell Signaling (#2535)
AMPKα	1:1000	Cell Signaling (#5832)
Phospho-ACC (Ser79)	1:1000	Cell Signaling (#3661)
ACC	1:1000	Cell Signaling (#3676)
FOXO1	1:1000	Cell Signaling (#9454)
PGC1β	1:1000	Abcam (ab176328)
E-cadherin	1:1000	Cell signaling (#3195)
Vimentin	1:1000	Cell signaling (#5741)
Snail	1:1000	Cell signaling (#3879)
β-Catenin	1:1000	Sigma-Aldrich (C7082)
β-Actin	1:20000	Sigma-Aldrich (A3854)
*E. coli* TnaA	1:2000	Assaypro (33517–05111)
β-Actin	1:20000	Sigma-Aldrich (A3854)
Anti-rabbit IgG, HRP-linked antibody	1:2000	Cell Signaling (#7074)
Anti-Mouse IgG, Peroxidase antibody	1:2000	Sigma-Aldrich (A9044)

**Table 4:** List of antibodies and conditions used in tissue microarray (TMA) analysis. AHR: aryl hydrocarbon receptor, PXR: pregnane X receptor.

Antibody	Dilution	Vendor
PXR	1:1000	Thermo Fisher Scientific (PA5–72551)
AHR	1:200	Sigma-Aldrich (HPA029722)

**Table 5:** Link between AHR or PXR expression and breast cancer patient survival. The effect of expression of AHR or PXR on survival in breast cancer was analyzed by kmplot.com, which is a freely accessible database. Total survival rates were assessed, and all samples are represented in different subpopulations of breast cancer. Numbers in bold represent statistically significant results.

**AHR (202820_at)**	**HR (Hazard Ratio)**	***p*-Value (Log Rank Test)**
All breast Cancers N = 3951	0.91	0.079
**ER(+), N = 3082**	**0.87**	**0.037**
ER(−), N = 869	0.96	0.68
PR(+), N = 589	0.72	0.068
PR(−), N = 549	0.87	0.35
HER2(+), N = 252	0.97	0.87
**HER2(−), N = 800**	**0.71**	**0.012**
ER(+), PR(+), HER2(+), N = 76	1.15	0.78
ER(−), PR(−), HER2(−), N = 198	0.84	0.48
**ER(+), PR(+), N = 577**	**0.67**	**0.029**
ER(−), PR(−), N = 298	0.84	0.39
ER(−), PR(−), HER2(−) N = 198	0.84	0.48
ER(+), Luminal A, N = 1933	0.87	0.11
ER(+), Luminal A, Grade 1, N = 267	0.65	0.17
ER(+), Luminal B, N = 1149	1.16	0.12
ER(+), Luminal B, Grade 1 N = 56	1.45	0.55
Grade 1, N = 345	0.67	0.13
**Grade 2, N = 901**	**0.67**	**0.001**
Grade 3, N = 903	0.97	0.78
Basal subtype, N = 618	0.95	0.67
Luminal A, N = 1933	0.87	0.11
Luminal B, N = 1149	1,16	0.12
ER(+), HER2(+), N = 156	1.33	0.36
ER(−), HER2(+), N = 96	0.67	0.21
**ER(+), PR(+), Lymph(+) N = 344**	**0.61**	**0.026**
ER(+), PR(+), Lymph(−) N = 228	0.86	0.64
ER(−), PR(−), Lymph(+) N = 127	0.85	0.54
ER(−), PR(−), Lymph(−) N = 167	0.68	0.21
**ER(+), Luminal A, Grade 2, N = 567**	**0.69**	**0.027**
ER(+), Luminal B, Grade 2 N = 253	0.76	0.21
**PXR (207202_s_at)**	**HR (Hazard Ratio)**	***p*-Value (Log Rank Test)**
**All breast Cancers N = 3951**	**0.8**	**0.00055**
**ER(+), N = 3082**	**0.84**	**0.0073**
**ER(−), N = 869**	**0.74**	**0.0056**
PR(+), N = 589	1.02	0.91
PR(−), N = 549	0.9	0.49
HER2(+), N = 252	0.93	0.73
HER2(−), N = 800	1.01	0.93
ER(+), PR(+), HER2(+), N = 76	1.31	0.61
ER(−), PR(−), HER2(−), N = 198	1.08	0.75
ER(+), PR(+), N = 577	0.97	0.89
ER(−), PR(−), N = 298	0.93	0.73
ER(−), PR(−), HER2(−) N = 198	1.048	0.75
**ER(+), Luminal A, N = 1933**	**0.77**	**0.0031**
ER(+), Luminal A, Grade 1, N = 267	1.04	0.9
**ER(+), Luminal B, N = 1149**	**0.81**	**0.031**
ER(+), Luminal B, Grade1 N = 56	0.77	0.66
Grade 1, N = 345	0.97	0.92
Grade 2, N = 901	1.07	0.56
Grade 3, N = 903	0.98	0.83
Basal subtype, N = 618	0.78	0.053
**Luminal A, N = 1933**	**0.77**	**0.0031**
**Luminal B, N = 1149**	**0.81**	**0.031**
ER(+), HER2(+), N = 156	1.41	0.28
ER(−), HER2(+), N = 96	0.63	0.15
ER(+), PR(+), Lymph(+) N = 344	0.96	0.85
ER(+), PR(+), Lymph(−) N = 228	0.84	0.6
ER(−), PR(−), Lymph(+) N = 127	0.95	0.86
ER(−), PR(−), Lymph(−) N = 167	1.02	0.95
ER(+), Luminal A, Grade 2, N = 567	1.06	0.73
ER(+), Luminal B, Grade2 N = 253	1.23	0.34

## Supplementary Materials

The following are available online at https://www.mdpi.com/2072-6694/12/9/2411/s1, the whole western blot images could be found in the supplementary materials.
