# Supplementary material for: Indolepropionic Acid, a Metabolite of the Microbiome, Has Cytostatic Properties in Breast Cancer by Activating AHR and PXR Receptors and Inducing Oxidative Stress
Source: Cancers (Basel). 2020 Aug 25;12(9):2411. doi: 10.3390/cancers12092411 (PMC7565149; doi:10.3390/cancers12092411)

3B

1. IPA CTL
2. IPA 0.4 µM
3. IPA 0.8 µM

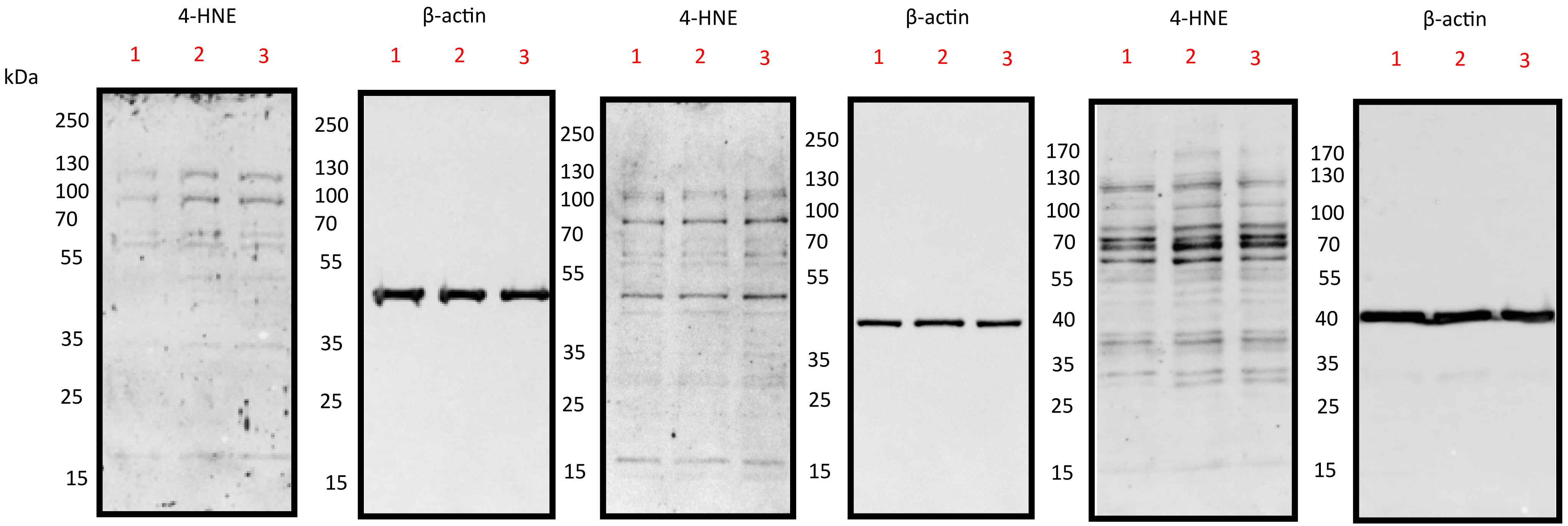

Ratio	FOLD1
0,178861201	1
0,214106228	1,197052391
0,221617371	1,239046648

Ratio	FOLD2
0,237951901	1
0,254600528	1,069966353
0,347017967	1,458353415

Ratio	FOLD3
0,218263588	1
0,265160436	1,214863363
0,257233793	1,178546525

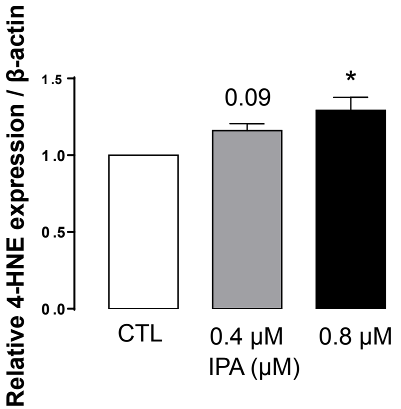

3C

1. IPA CTL
2. IPA 0.4 μM
3. IPA 0.8 μM

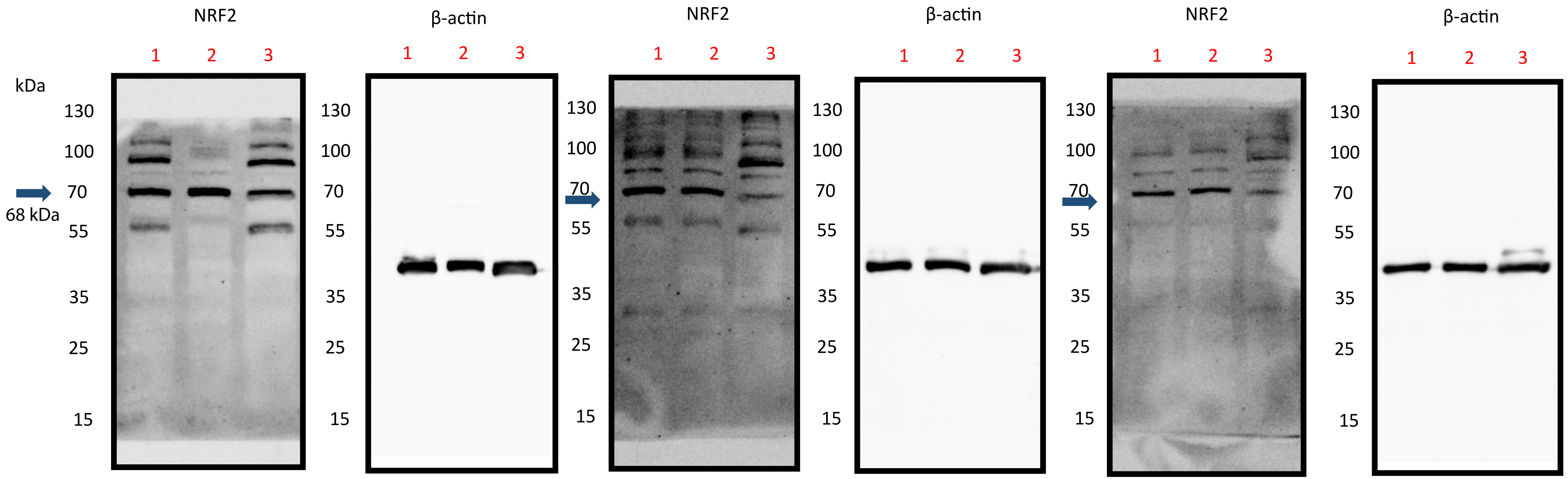

Ratio	FOLD1
1,087727275	1
0,977136786	0,898328845
0,927427741	0,852628929

Ratio	FOLD2
1,030933889	1
0,957980575	0,929235701
0,733379495	0,711373933

Ratio	FOLD3
0,950708312	1
0,826014167	0,868840797
0,663676982	0,698086861

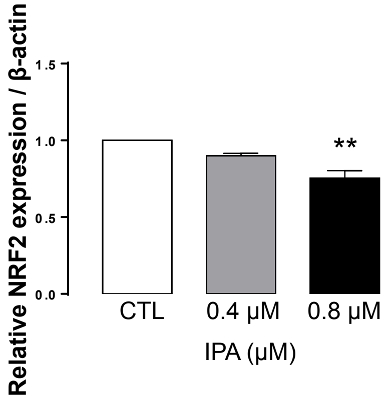

3C

1. IPA CTL
2. IPA 0.4 µM
3. IPA 0.8 µM

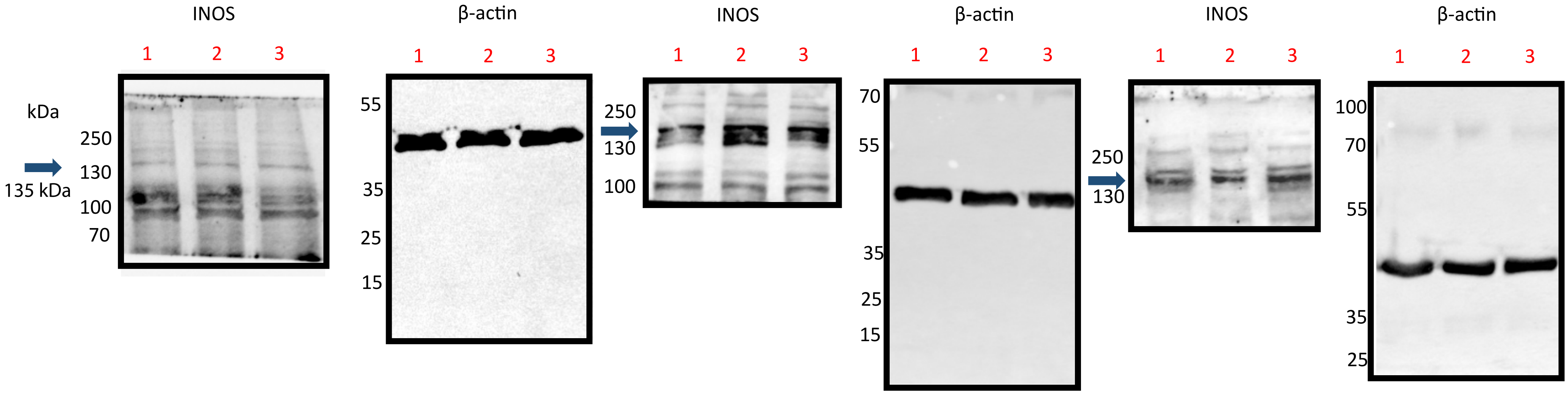

Ratio	FOLD1
0,226876575	1
0,252211628	1,111668879
0,288036175	1,269572123

Ratio	FOLD2
0,609317504	1
0,93330395	1,531720233
1,077912258	1,769048567

Ratio	FOLD3
0,430557542	1
0,496148098	1,152338652
0,570800492	1,325724057

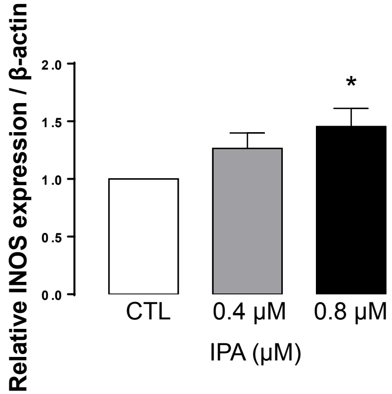

3E

1. IPA CTL
2. IPA 0.4 μM
3. IPA 0.8 μM

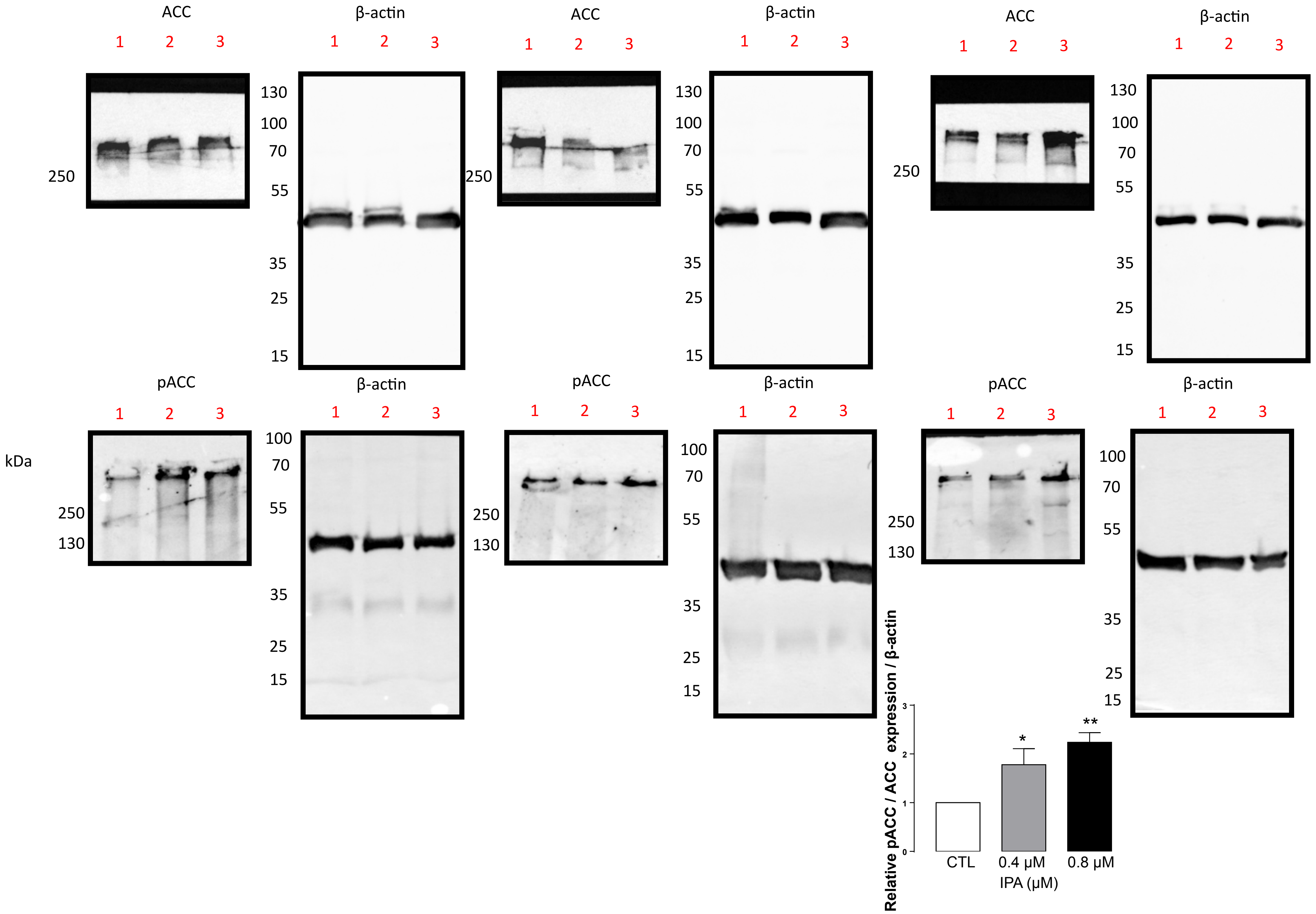

3E

1. IPA CTL
2. IPA 0.4 µM
3. IPA 0.8 µM

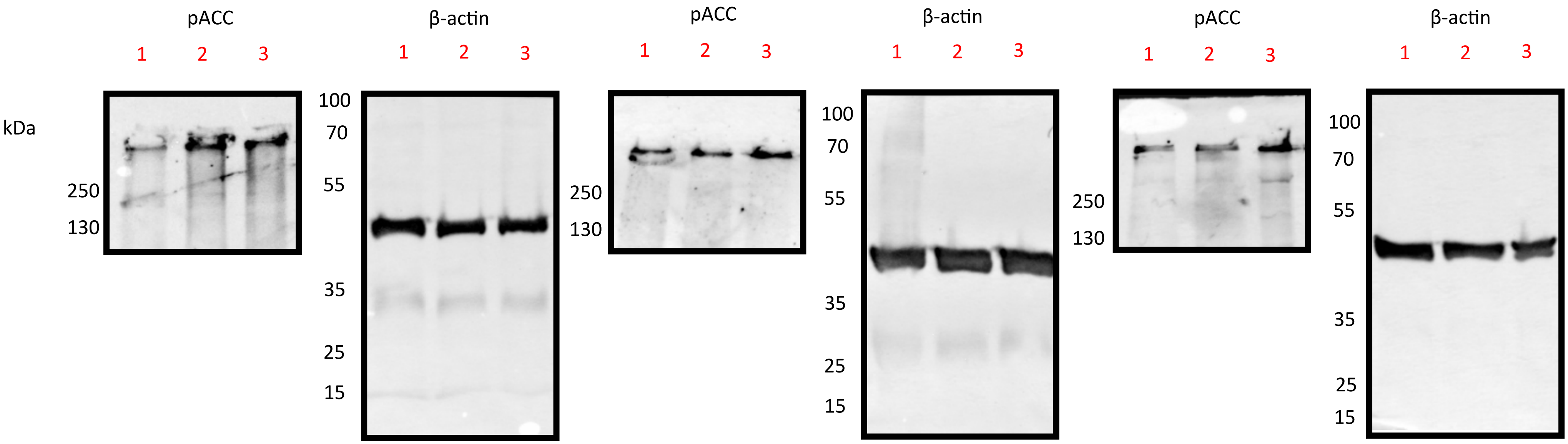

Ratio	**FOLD1**
0,465462949	1
0,952987061	2,047396172
0,947433086	2,03546402

Ratio	**FOLD2**
0,861885354	1
0,907392149	1,052799128
1,173240989	1,361249479

Ratio	**FOLD3**
0,584047919	1
0,636127195	1,089169527
1,528026736	2,616269464

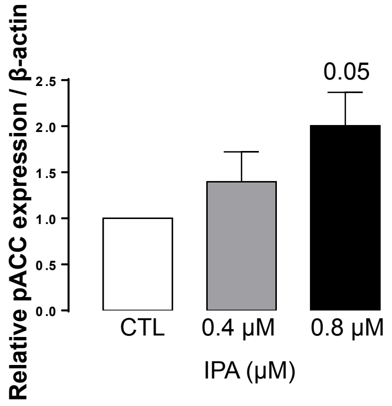

3E

1. IPA CTL
2. IPA 0.4 µM
3. IPA 0.8 µM

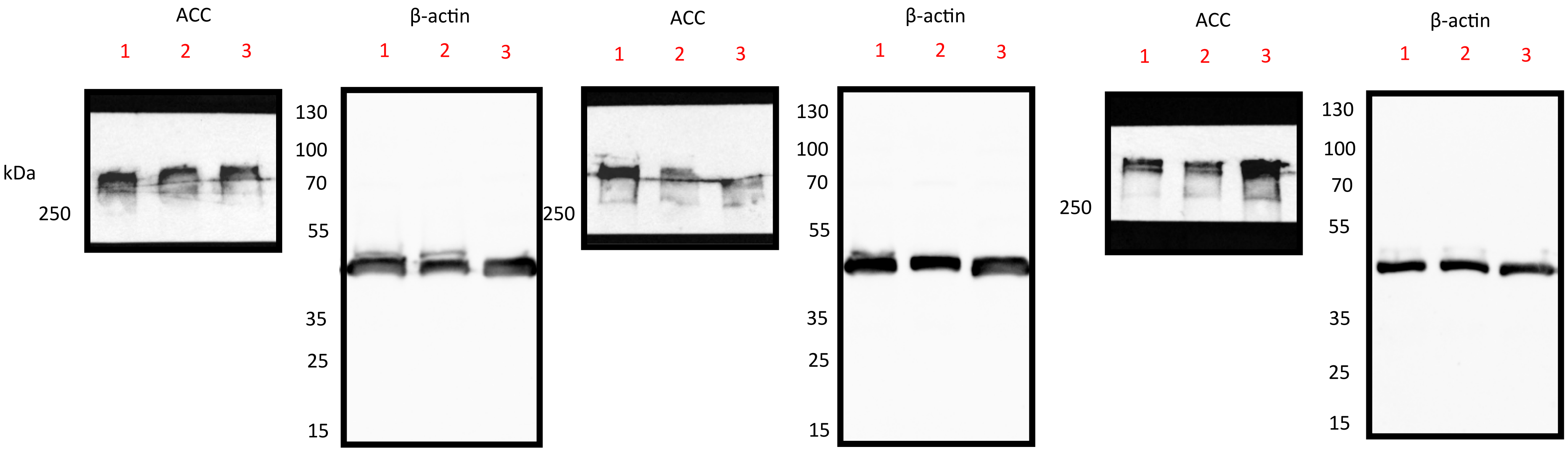

Ratio	FOLD1
1,000795267	1
0,845981297	0,84530905
0,879390267	0,878691472

Ratio	FOLD2
0,840540218	1
0,555661132	0,661076199
0,613099254	0,729410968

Ratio	FOLD3
0,938664799	1
0,773123204	0,823641415
0,968404372	1,031682847

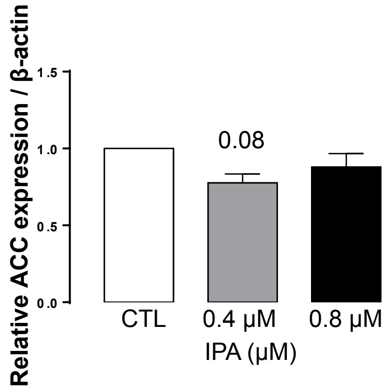

3E

1. IPA CTL
2. IPA 0.4 μM
3. IPA 0.8 μM

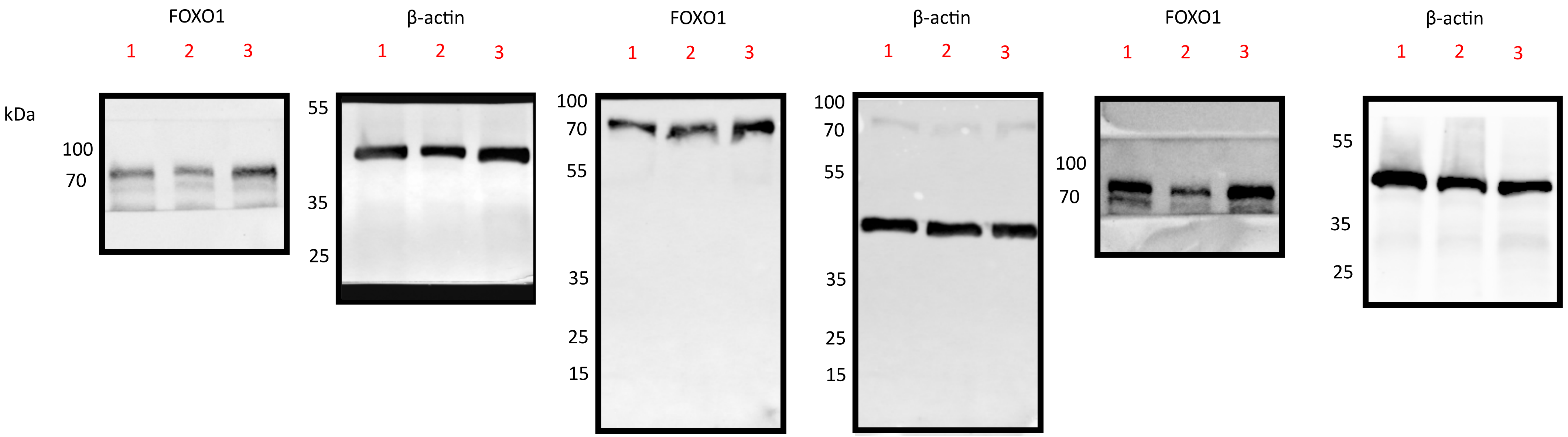

Ratio	FOLD1
0,437623304	1
0,492109349	1,124504441
0,771326809	1,762535957

Ratio	FOLD2
0,543568572	1
0,648662035	1,193339843
0,972191738	1,788535592

Ratio	FOLD3
1,002604112	1
1,036991281	1,034297853
1,408209586	1,404551975

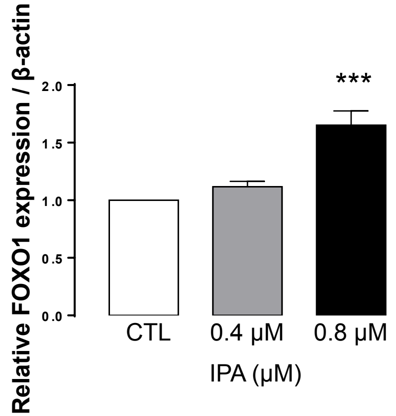

3E

1. IPA CTL
2. IPA 0.4 μM
3. IPA 0.8 μM

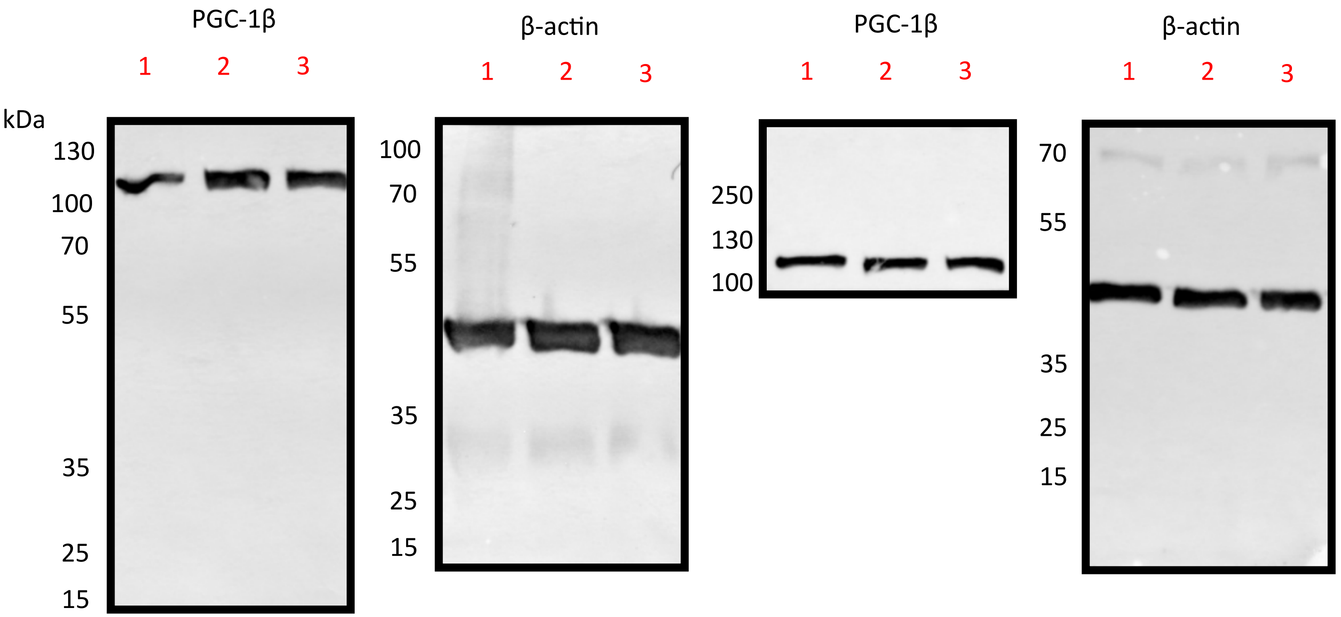

Ratio	FOLD1
0,787472713	1
1,179511228	1,497843937
1,044594011	1,326514551

Ratio	FOLD2
0,693513303	1
0,839986099	1,211204017
0,934049756	1,346837547

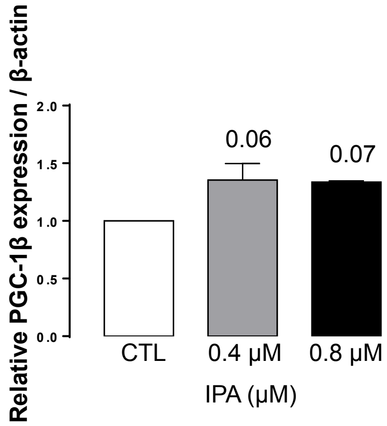

4D

1. IPA CTL
2. IPA 0.4 µM
3. IPA 0.8 µM

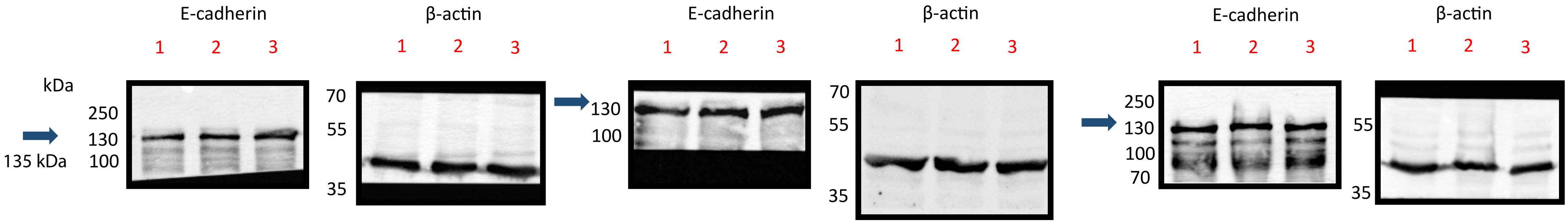

Ratio	**FOLD1**
0,630159589	1
0,802025508	1,272733958
0,827998862	1,313951063

Ratio	**FOLD2**
0,92570575	1
1,122243536	1,212311295
1,201059178	1,297452434

Ratio	**FOLD3**
0,680733772	1
0,857461681	1,259613841
0,948386537	1,393182733

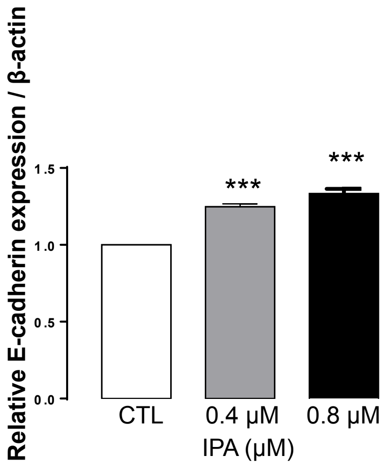

4D

1. IPA CTL
2. IPA 0.4 µM
3. IPA 0.8 µM

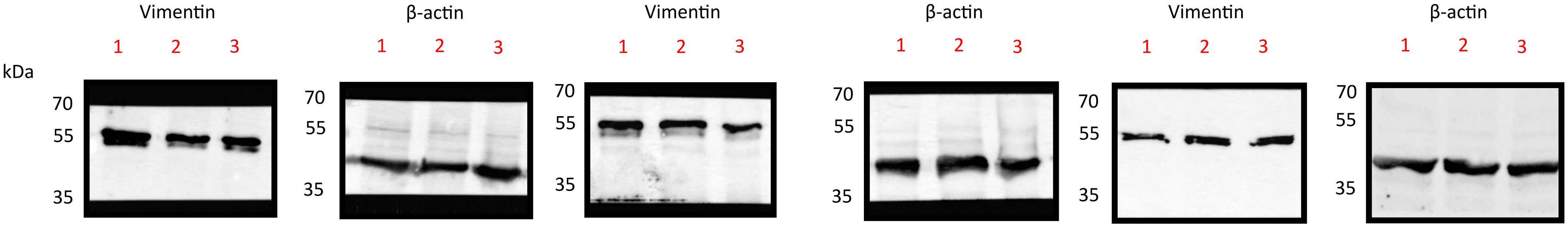

Ratio	FOLD1
1,248302602	1
0,980542361	0,785500535
0,804445737	0,644431676

Ratio	FOLD2
0,941890991	1
0,874886934	0,928862195
0,735788655	0,781182389

Ratio	FOLD3
1,018470955	1
0,836426148	0,821256752
0,855836197	0,840314781

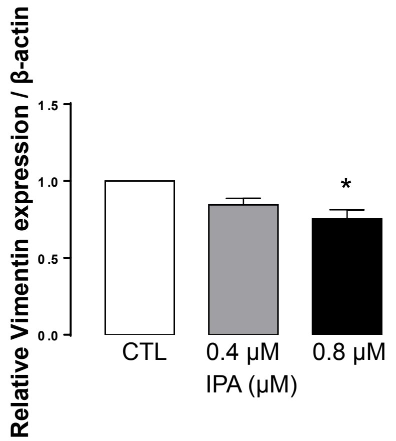

4D

1. IPA CTL
2. IPA 0.4 μM
3. IPA 0.8 μM

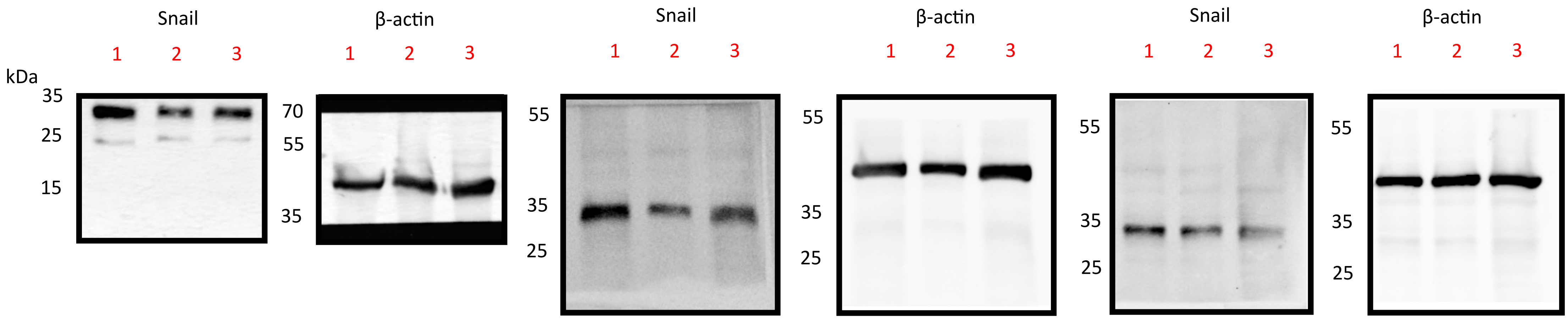

Ratio	FOLD1
1,002487904	1
0,583092174	0,581645097
0,641425978	0,639834132

Ratio	FOLD2
1,205302039	1
0,725928788	0,602279565
0,688672592	0,571369308

Ratio	FOLD3
0,803377018	1
0,597539559	0,74378473
0,471320377	0,586673961

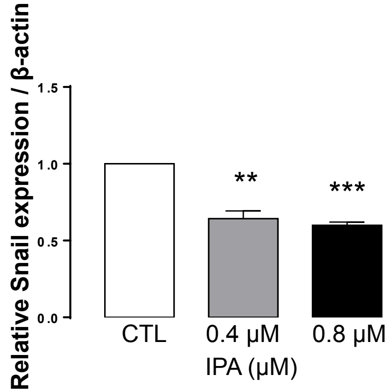

4D

1. IPA CTL
2. IPA 0.4 µM
3. IPA 0.8 µM

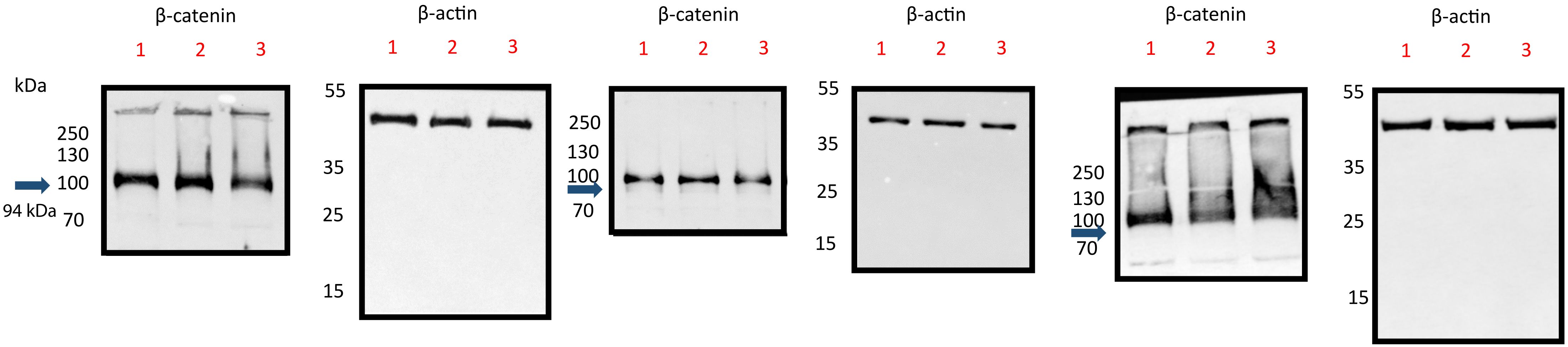

Ratio	FOLD1
1,046949086	1
1,009968381	0,964677647
0,832058294	0,794745709

Ratio	FOLD2
0,884683255	1
0,758626698	0,857512216
0,731012592	0,826298665

Ratio	FOLD3
0,906775503	1
0,712971349	0,786271074
0,71547299	0,789029906

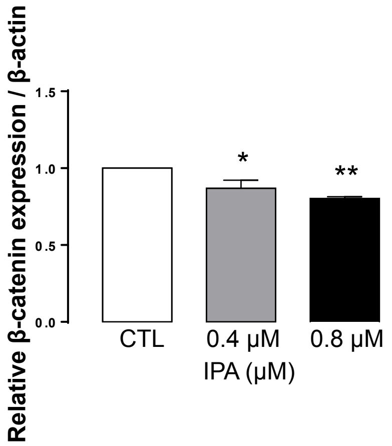

6B

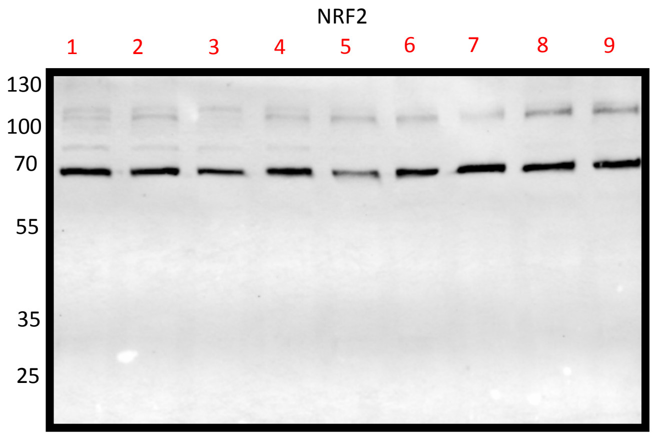

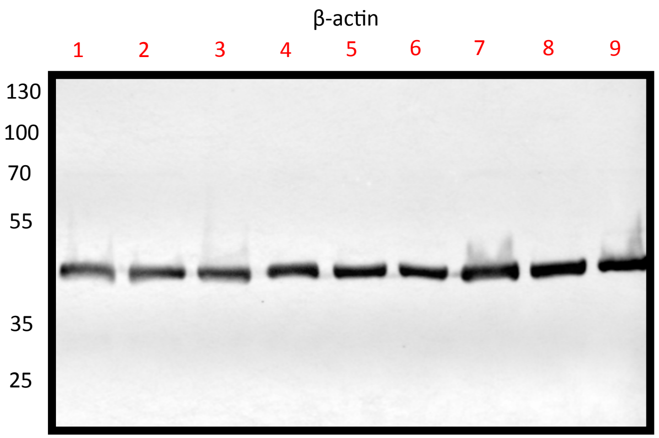

Ratio	FOLD1
1,305741555	1
1,1679876	0,894501362
0,88128724	0,674932368
0,931209389	1
0,537212341	0,576897471
0,822640679	0,883411066
0,937215229	1
0,85440254	0,911639626
0,833168692	0,888983305

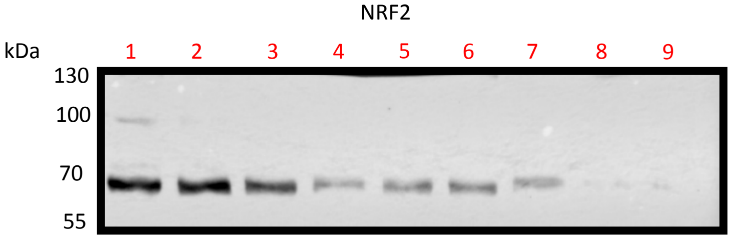

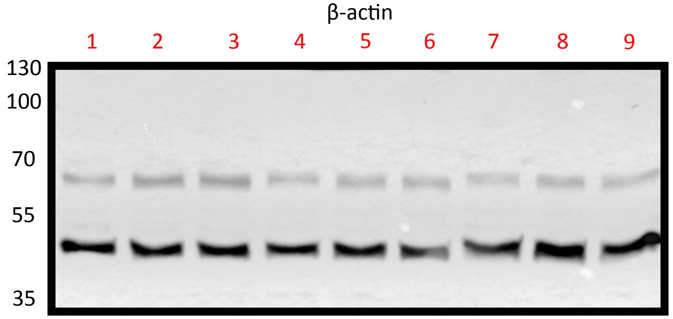

Ratio	FOLD2
1,086096421	1
0,964744341	0,888267673
0,674151779	0,620710801
0,408311869	1
0,467825309	1,145754862
0,618944535	1,515862219
0,338837366	1
0,192926686	0,569378426
0,222110677	0,655508214

1. IPA CTL
2. IPA 0.4 μM
3. IPA 0.8 μM
4. IPA CTL
5. IPA 0.4 μM
6. IPA 0.8 μM
7. IPA CTL
8. IPA 0.4 μM
9. IPA 0.8 μM

(4–6: CH223191 (AHRi); 7–9: Ketoconazole (PXRi))

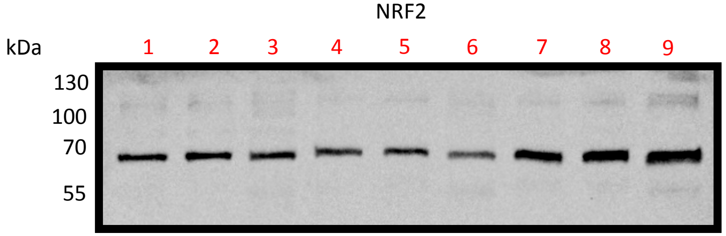

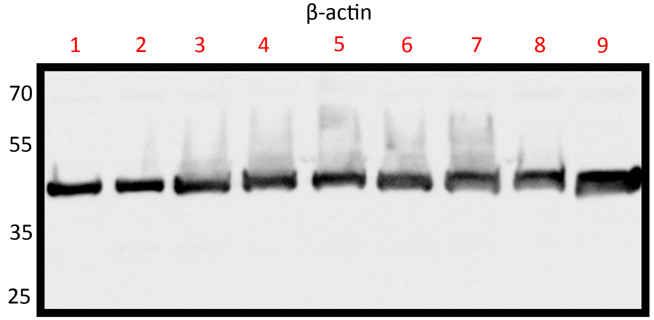

Ratio	FOLD3
0,989696377	1
0,894384523	0,903695864
0,861196668	0,870162493
0,869978269	1
0,846534534	0,973052506
0,779752216	0,896289303
1,247683352	1
1,283337424	1,028576219
1,067683846	0,855733022

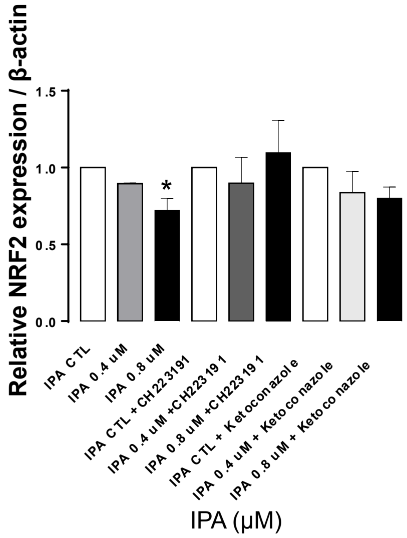

6B

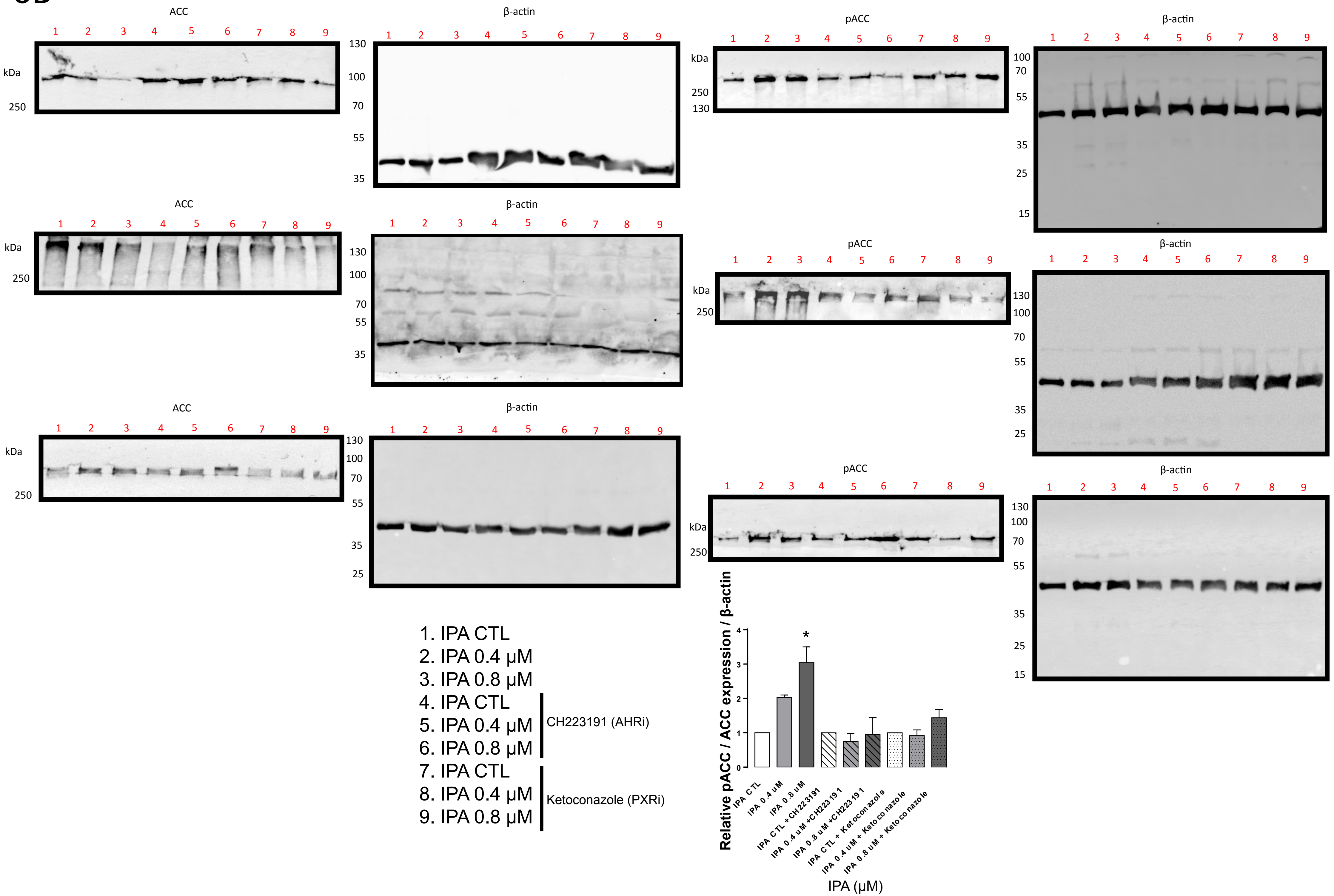
ACC
β-actin
pACC
kDa
1 2 3 4 5 6 7 8 9
250
130
100
70
55
35
25
15
1. IPA CTL
2. IPA 0.4 μM
3. IPA 0.8 μM
4. IPA CTL
5. IPA 0.4 μM
6. IPA 0.8 μM
CH223191 (AHRi)
7. IPA CTL
8. IPA 0.4 μM
9. IPA 0.8 μM
Ketoconazole (PXRi)
Relative pACC / ACC expression / β-actin
0
1
2
3
4
*
IPA CTL
IPA 0.4 uM
IPA 0.8 uM
IPA CTL + CH223191
IPA 0.4 uM +CH223191
IPA 0.8 uM +CH223191
IPA CTL + Ketoconazole
IPA 0.4 uM + Ketoconazole
IPA 0.8 uM + Ketoconazole
IPA (μM)

6B

pACC

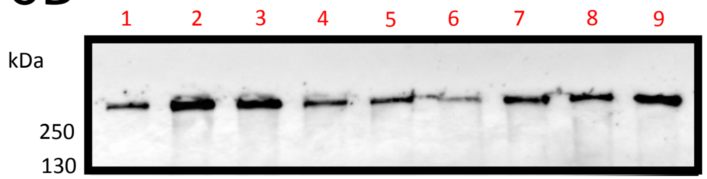

β-actin

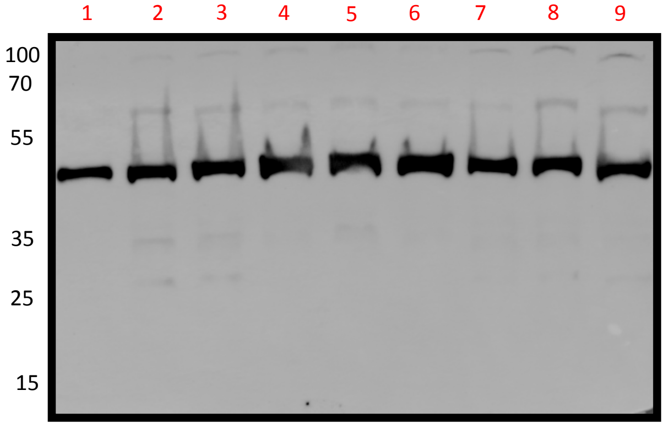

Ratio	FOLD1
0,702269187	1
1,017160393	1,448391034
1,021721811	1,45488629
0,88327815	1
0,847579776	0,959584221
0,417179604	0,472308303
0,900628727	1
0,904873546	1,004713173
0,945056703	1,049329957

pACC

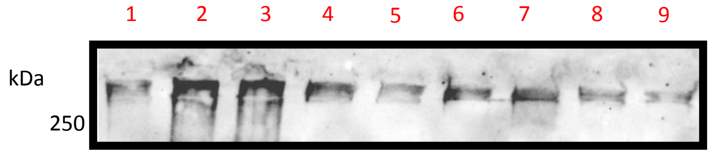

β-actin

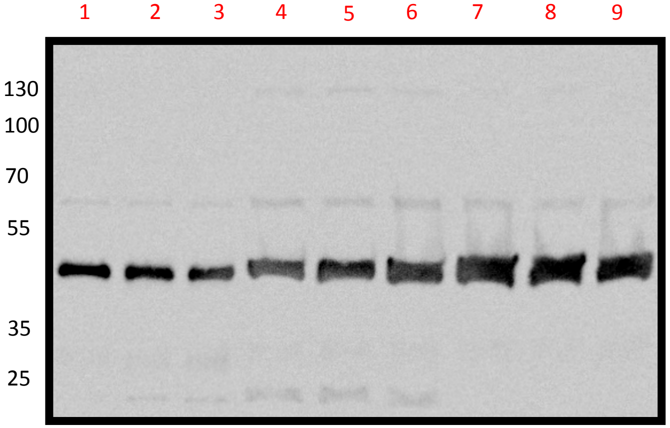

Ratio	FOLD2
0,437835177	1
0,776514644	1,773531878
0,897651574	2,050204326
0,875128363	1
0,47188498	0,539218017
0,672282514	0,768210176
0,626000323	1
0,408795956	0,653028346
0,373547325	0,596720658

1. IPA CTL
2. IPA 0.4 µM
3. IPA 0.8 µM
4. IPA CTL
5. IPA 0.4 µM
6. IPA 0.8 µM
7. IPA CTL
8. IPA 0.4 µM
9. IPA 0.8 µM

CH223191 (AHRi) (4–6)

Ketoconazole (PXRi) (7–9)

pACC

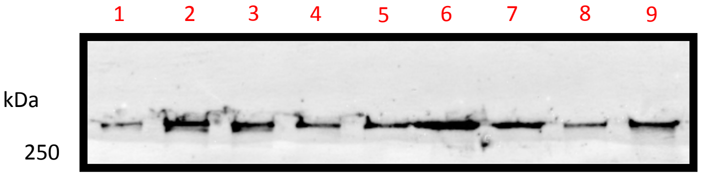

β-actin

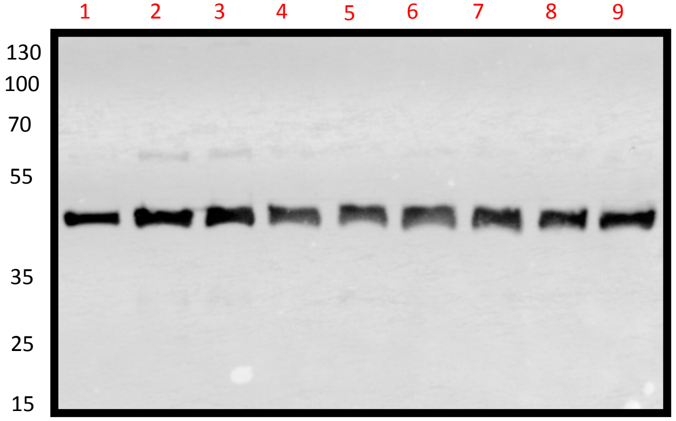

Ratio	FOLD3
0,38304045	1
0,862150889	2,25080899
1,000777809	2,61272095
0,909831633	1
0,964016154	1,059554448
1,328701137	1,460381337
0,822836466	1
0,609014582	0,740140487
0,75856018	0,921884495

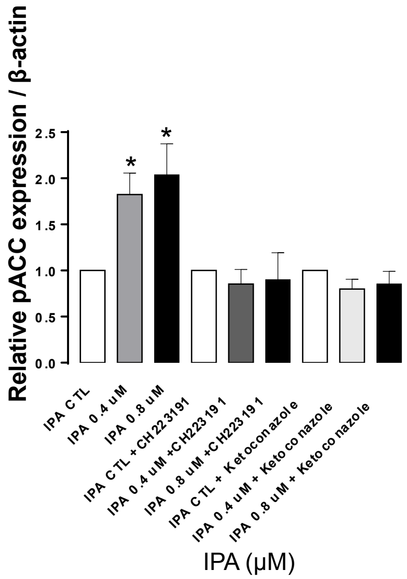

6B

ACC

1 2 3 4 5 6 7 8 9

kDa

250

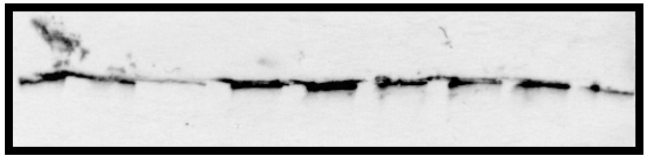

β-actin

1 2 3 4 5 6 7 8 9

130
100
70
55
35

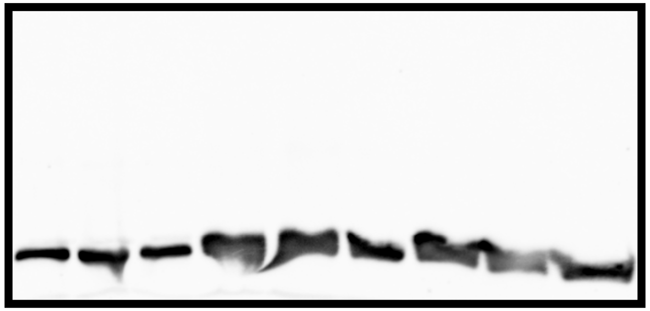

Ratio	FOLD1
0,640337916	1
0,459197062	0,717116777
0,251196269	0,392287046
0,815996679	1
0,9998635	1,225327904
0,737702657	0,904051053
0,67667251	1
1,160387938	1,714844212
0,421742689	0,623259676

ACC

1 2 3 4 5 6 7 8 9

kDa

250

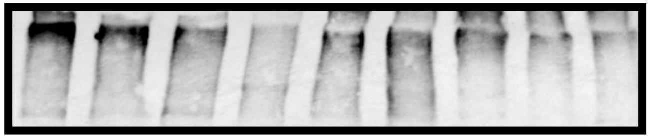

β-actin

1 2 3 4 5 6 7 8 9

130
100
70
55
35

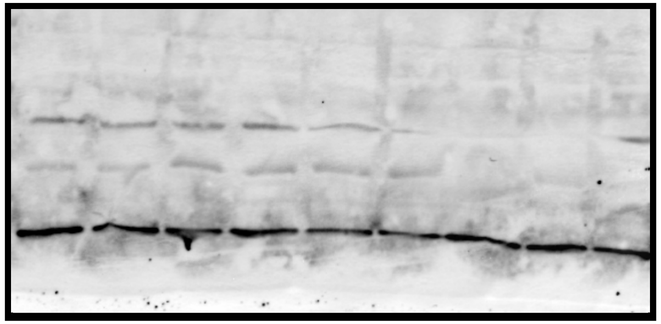

Ratio	FOLD2
1,276539125	1
1,188065951	0,93069294
0,802506897	0,628658285
0,346103351	1
0,570559206	1,648522629
0,709942599	2,051244512
0,82287736	1
0,503790001	0,612229751
0,508209459	0,617600488

1. IPA CTL
2. IPA 0.4 µM
3. IPA 0.8 µM
4. IPA CTL
5. IPA 0.4 µM
6. IPA 0.8 µM
7. IPA CTL
8. IPA 0.4 µM
9. IPA 0.8 µM

4–6: CH223191 (AHRi)

7–9: Ketoconazole (PXRi)

ACC

1 2 3 4 5 6 7 8 9

kDa

250

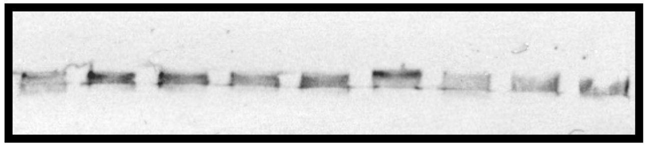

β-actin

1 2 3 4 5 6 7 8 9

130
100
70
55
35
25

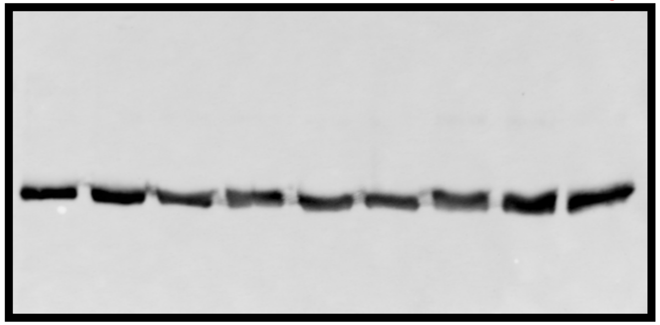

Ratio	FOLD3
0,961104555	1
1,000892106	1,041397734
1,171371062	1,218775893
1,108723148	1
1,037637486	0,93588511
0,832048558	0,750456558
0,832259773	1
0,557153398	0,669446507
0,460129816	0,552868024

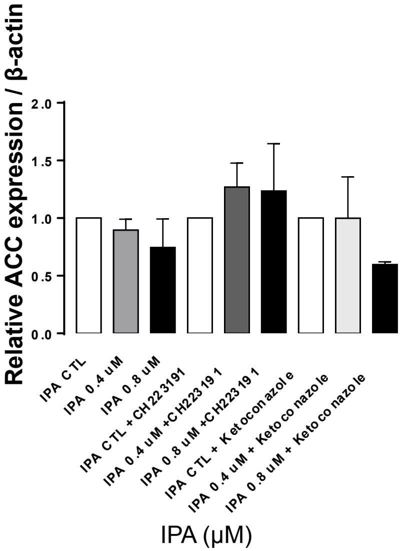

Supplement: Supplementary file 1 [file cancers-12-02411-s001.pdf]
